# Large deviations of piecewise-deterministic Markov processes with application to stochastic calcium waves

**DOI:** 10.1007/s00285-026-02446-7

**Published:** 2026-08-28

**Authors:** Gaetan Barbet, James MacLaurin, Moshe Silverstein, Pedro Vilanova

**Affiliations:** 1 Child Health Institute of New Jersey, Department of Pediatrics and Department of Pharmacology, Robert Wood Johnson Medical School, Rutgers University, New Brunswick, USA; 2 Department of Mathematics, Stevens Institute of Technology, Hoboken, USA; 3 Department of Mathematical Sciences, New Jersey Institute of Technology, Newark, USA

**Keywords:** 2020, 60J25, 92C37

## Abstract

We prove a large deviation principle for piecewise deterministic Markov processes (PDMPs). This is an asymptotic estimate for the probability of a trajectory in the large size limit. Explicit Euler–Lagrange equations are determined for computing optimal first-hitting-time trajectories. The results are applied to a model of stochastic calcium dynamics. It is widely conjectured that the mechanism of calcium puff generation is a multiscale process: with microscopic stochastic fluctuations in the opening and closing of individual channels generating cell-wide waves via the diffusion of calcium and other signaling molecules. We model this system as a PDMP, with N≫1 stochastic calcium channels that are coupled via the ambient calcium concentration. We employ the large deviations theory to estimate the probability of cell-wide calcium waves being produced through microscopic stochasticity.

This paper determines a large deviation principle for piecewise-deterministic Markov processes (PDMPs), in the large population size limit. Piecewise-deterministic Markov processes consist of a Markov Chain flipping between discrete states, coupled to an ordinary differential equation that evolves deterministically in-between the flips (and whose evolution typically depends on the state of the Markov Chain) (Hasler et al. 2013; Azaïs and Muller-Gueudin 2016). PDMPs are often used to model slow/fast systems (the ODEs typically correspond to stochastic equations that rapidly equilibrate) (Bressloff and Maclaurin 2018). PDMPs enjoy numerous applications, particularly in biology (Goldwyn and Shea-Brown 2011; Rudnicki and Tyran-Kaminska 2017; Bressloff and Maclaurin 2018; Bressloff 2021a, b). They have been used (for instance) to model excitable membranes in neuroscience (Riedler et al. 2012; Lawley et al. 2016), population dynamics in ecology (Hening et al. 2023), run-and-tumble dynamics of bacteria (Berg 1993) and stochastic models of calcium signals (Keizer et al. 1998; Ramlow et al. 2023a; Ramlow, Falcke and Lindner 2023b).

Often, one wishes to estimate the probability of rare-events in PDMPs (Kim et al. 2022). For example, one may be interested in the probability of extinction of a species in population dynamics (Peliti 1985) or rare events in cellular stochasticity (Bressloff and Newby 2014), or the spontaneous and stochastic production of waves / pulses (Lecar and Nossal 1971; Chow and White 1996; Keener and Newby 2011; Newby 2014). A well-known biological example-which is thoroughly explored in this paper-is stochastic calcium dynamics (this will be discussed in more detail in Sect. 3 below) (Rüdiger 2014).

One of the best tools for estimating the probability of rare events is the theory of large deviations (Budhiraja and Dupuis 2019; Avitabile and MacLaurin 2026). To the authors’ knowledge, the first scholar to study the large deviations of PDMPs was Kifer (2009). This was followed by the work of Faggionato et al. (2009), who determined a large deviation principle for piecewise-deterministic Markov Processes. The model of Faggionato et al is different from ours: in their model the Markov Chain flips asymptotically quickly on a fixed discrete space, whereas in our model as $N\rightarrow \infty$ the size of the flips scales as $N^{-1}$, and the rate scales as *N*. Bressloff and Faugeras (2017) studied a very similar model to Faggionato et al, and demonstrated that the large deviations rate function can be written to have a Hamiltonian structure (under an assumption of ergodicity). Our model does not require any assumption of ergodicity. In fact this is relevant to many biological applications, because (for example) extinction is usually an important possibility.

An interesting biological application that involves large deviations of PDMPs can be found in the recent work of Keener and Newby (2011), and Newby (2014). These papers concern the spontaneous production of action potentials in the stochastic Morris-Lecar Model. Newby (2014) employed large deviation theory to compute the quasi-potential for the system (the quasi-potential yields the optimal means that the system produces an action-potential, by first taking the system size $N\rightarrow \infty$, then $T\rightarrow \infty$ (Freidlin and Wentzell 2012)). He also explicitly outlined the Euler–Lagrange equations that give the optimal trajectory, just like this paper. One of the main conclusions of Newby was that characterizing noise-driven action potentials using a fast/slow separation overlooks the possibility that noise in the slow subsystem, not the fast subsystem, is the main contributor to spontaneous action potentials.

On a related note, there has been much recent interest in the large deviations of chemical reaction networks (Patterson and Renger 2019). These are high-dimensional jump Markovian Processes, similar to the PDMPs of this paper, except without the ODE dynamics. Recent works include those of Dupuis et al. (2016), Pardoux and Samegni-Kepgnou (2017), Agazzi et al. (2018), and Patterson and Renger (2019). Patterson and Renger (2019) and Agazzi, Andreis, Patterson and Renger Agazzi,Andreis and Patterson (2022) prove the large deviations principle in a general setting by also studying the convergence of the reaction fluxes (like in this paper). Budhiraja and Friedlander (2018) determine the large deviations of jump-Markov processes. There is also a related literature on the large deviations of slow-fast systems (Kumar and Popovic 2017; Kraaij and Schlottke 2020).

One of the novelties of the proof in this paper is its simplicity. We map the PDMP system to a set of homogeneous Poisson Processes via a time-rescaling. The large deviation principle is then an almost immediate result of the Inverse Contraction Principle (Dembo and Zeitouni 1998). In addition, we explicitly determine the structure of the Euler–Lagrange equations (needed for computing optimal first-hitting times). This becomes an optimization problem over the time interval [0, *T*], subject to a constraint (given by the slow processes). We then solve these equations to compute the probability of noise-induced calcium waves.

## Overview of stochastic models of calcium signaling

In Sect. 3 we apply our large deviations result to a stochastic model of the production of calcium waves in cells. Classical models of calcium signalling are almost entirely deterministic (Keener 2006). These models typically assumed that the calcium concentration (and signalling molecules such as IP3) within the cell can be well-approximated as homogeneous, and therefore the dynamics can be accurately described by ordinary differential equations for the evolution of their concentrations in time (Dupont et al. 2016). However recent experimental evidence / mathematical analysis has called this into question (Keizer and Smith 1998; Keener 2006; Skupin et al. 2008; Keener and Sneyd 2008; Moenke et al. 2012; Rüdiger 2014; Ramlow et al. 2023a; Ramlow, Falcke and Lindner 2023b). It has been demonstrated that (i) the interspike interval can show significant variability, and (ii) intracellular calcium concentrations show steep gradients, and therefore it is widely postulated that much of the emergent phenomena are stochastic in nature (Ramlow et al. 2023a; Ramlow, Falcke and Lindner 2023b).

One of our fundamental aims is to develop an accurate microscopic model of calcium signalling (which is thoroughly stochastic) and then use statistical mechanical techniques to determine effective macroscopic equations. Calcium signaling is organized in a hierarchical manner (Cao et al. 2013; Dupont et al. 2016; Friedhoff et al. 2021). At the lowest (most microscopic) level, stochastic release of $Ca^{2+}$ upon binding of IP3 receptor results in a small localized increase in cytoplasmic $Ca^{2+}$ concentration, which is often termed a ‘blip’. The IP3 receptors are typically tightly clustered, which means that a $Ca^{2+}$ blip can stimulate the release of additional $Ca^{2+}$ through neighboring receptors, so that the entire cluster emits a localized ‘puff’. At the highest level of organization, if enough puffs are generated, they can form a propagating wave of increased $Ca^{2+}$ across an entire cell. There is considerable evidence that calcium puffs and waves are nonlinear stochastic phenomena: it has been observed for instance that IP3 channels can be highly active even when the average open probability is less than half its maximum (Siekmann et al. 2012). The very presence of abortive calcium waves is indicative of the stochastic origins of the phenomena. Indeed clusters have a diameter of 60–100 nm whereas the distance between clusters is much larger: 3–7 micrometers (Dupont et al. 2016).

There is a well-established literature that examines how spatially-distributed calcium waves and patterns can arise out of microscopic stochastic models. Some of the earliest work is in the fire–diffuse–fire model of Keizer et al. (1998); Keizer and Smith (1998). In this model, Ryanodine Receptors open (and release calcium) once the ambient calcium exceeds a threshold; before closing and entering a refractory state before being ready to open again. Keizer and Smith were able to demonstrate that this model exhibits spatially-distributed waves. Coombes and Timofeeva (2003); Coombes et al. (2004) developed a similar model. They estimated the release probability for a cluster of channels and determined that it is approximately a sigmoidal function of the local calcium concentration. Keener (2006) further extended these works by taking the opening and closing of individual channels to be stochastic. Hinch and Chapman (2005) used exponential asymptotic methods to determine the relative frequency of calcium sparks. Falcke et al employed approximations to determine estimates for the approximate probability of calcium puffs (given the frequency of blips), and also estimates for the probability of a wave throughout the cell (Moenke et al. 2012; Dupont et al. 2016). An advantage of all of the previous works is that the models are all spatially extended, unlike this paper. However the model in this paper fully couples the calcium concentration to the opening / closing of the channel. It thus allows a more detailed estimation of how the feedback between the opening / closing of channels and the local calcium concentration leads to calcium waves.

This paper is structured as follows. In Sect. 1, we introduce the model and outline our main results. In Sect. 2, we determine the Euler–Lagrange equations that follow from the large deviation principle. These can be used to estimate optimal trajectories for first-hitting-time problems. In Sect. 3 we apply the theory to estimate the probability of the spark-to-wave transition in calcium dynamics. Finally in Sect. 4 we outline the proofs.

*Notation:*

Let $\left\| \cdot \right\| _t$ denote the supremum norm over [0, *t*], i.e.

$$ \left\| z \right\| _t = \sup _{s\in [0,t]}|z_s|. $$

Let $D([0,t],\mathbb {R})$ be the cadlag space of real functions from [0, *t*] to $\mathbb {R}$ that are right-continuous and have left-hand limits. Define $\Lambda _t$ to be the class of strictly increasing, continuous mappings of [0, *t*] onto itself (so that any $\lambda \in \Lambda _t$ is such that λ(0)=0 and λ(t)=t. Following Billingsley (1999), for any $\lambda \in \Lambda$, define

1 $$\begin{aligned} \left\| \lambda \right\| ^{\circ }_t =\sup _{s< t}\bigg |\log \frac{\lambda (t) - \lambda (s)}{t-s} \bigg | \end{aligned}$$

Define the Skorohod Metric on $\mathcal {D}([0,t], \mathbb {R})$,

2 $$\begin{aligned} d^{\circ }_t(x,y) = \inf _{\lambda \in \Lambda _t} \big \lbrace \left\| \lambda \right\| ^{\circ }_t \wedge \sup _{s\in [0,t]}| x(s) - y(\lambda (s)) | \big \rbrace . \end{aligned}$$

It is proved in Billingsley (1999) that $d^{\circ }_t$ is a metric, and that $\mathcal {D}([0,t], \mathbb {R})$ is complete and separable with respect to the topology induced by $d^{\circ }_t$. Next, for $a\in \mathbb {Z}^+$ define the pseudo-metric $\tilde{d}^{\circ }_a: \mathcal {D}([0,\infty ),\mathbb {R})\times \mathcal {D}([0,\infty ),\mathbb {R}) \rightarrow \mathbb {R}^+$ to be

3 $$\begin{aligned} \tilde{d}^{\circ }_a(x,y) =&d^{\circ }_a( g_a x, g_a y) \text { where }\end{aligned}$$

4 $$\begin{aligned} g_a(t) =&\left\{ \begin{array}{c} 1 \text { if }t\le a-1 \\ 0 \text { if }t\ge a \\ a-t \text { if }a-1 \le t \le a \end{array} \right. \end{aligned}$$

Then define the metric $d^{\circ }_{\infty }: \mathcal {D}([0,\infty ),\mathbb {R})\times \mathcal {D}([0,\infty ),\mathbb {R}) \rightarrow \mathbb {R}^+$ to be

5 $$\begin{aligned} d^{\circ }_{\infty }(x,y) = \sum _{a=1}^{\infty } \inf \big \lbrace 2^{-a} , \tilde{d}^{\circ }_a(x,y) \big \rbrace . \end{aligned}$$

It is proved in Billingsley (1999) that $d^{\circ }_{\infty }$ metrizes convergence over $\mathcal {D}([0,\infty ),\mathbb {R})$. Let

6 $$\begin{aligned} \tilde{\mathcal {D}}([0,\infty ),\mathbb {R}^+) \subset \mathcal {D}([0,\infty ),\mathbb {R}^+) \end{aligned}$$

consist of all paths that (i) start at 0, (ii) are non-decreasing.

## Model outline and large deviations principle

A chemical reaction network (Anderson and Kurtz 2015) consists of the following triple:

- *Species* $\mathscr {S}$, which are the chemical components whose counts we wish to model dynamically. There are *d* of these, and thus the species count can be represented by a vector in $\mathbb {Z}^d_+$.
- *Complexes* $\mathscr {C}$ which are nonnegative linear combinations of the species that describe how the species can interact and
- *Reactions* $\mathscr {R}$ which describe how to convert one complex to another. There are *M* of these.

Let $X(t) \in \mathbb {Z}_+^d$ be a vector that counts the number of particles of each species. For a reaction $\alpha \in \mathscr {R}$, let $Z_{\alpha }(t) \in \mathbb {Z}_+$ count the number of times the reaction has occurred. We thus have that

7 $$\begin{aligned} X(t) =X(0) + \sum _{\alpha \in \mathscr {R}}Z_{\alpha }(t) \xi _{\alpha } \end{aligned}$$

where $\xi _\alpha \in \mathbb {Z}^d$ is the reaction vector corresponding to the $\alpha ^{th}$ reaction. We define the concentration vector to be the scaled number of particles, i.e.

8 $$\begin{aligned} x(t)&= N^{-1} X(t) = N^{-1}X(0)+ \sum _{\alpha \in \mathscr {R}}z_{\alpha }(t) \xi _{\alpha } , \end{aligned}$$

where N≫1 is a scaling parameter that indicates the system size (for instance, the total number of particles at time), and $z_{\alpha }(t) = N^{-1}Z_{\alpha }(t)$.

The intensity of the $\alpha ^{th}$ reaction is defined to be

9 $$\begin{aligned} N\lambda _\alpha \big ( x(t) , u(t) \big ), \end{aligned}$$

where $\lambda _\alpha : \mathbb {R}^d \times \mathbb {R}^m \rightarrow \mathbb {R}^+$ is continuous and bounded. It is assumed that reactions cannot produce a negative concentration: that is, it is assumed that if $x\in \mathbb {R}_+^d$ and $u \in \mathbb {R}^m$, and $\alpha \in \mathscr {R}$ are such that $x + N^{-1}\xi _{\alpha } \notin \mathbb {R}_+^d$, then necessarily $\lambda _{\alpha }(x,u) =0$.

The ordinary differential equation takes values in a state space $\mathbb {R}^m$, and is such that

10 $$\begin{aligned} \frac{du}{dt} = A\big ( u(t), x(t) \big ), \end{aligned}$$

where $A: \mathbb {R}^m \times \mathbb {R}^d \rightarrow \mathbb {R}^d$ is continuous. The initial conditions are taken to be non-random constants i.e.

11 $$\begin{aligned} u(0)&:= \hat{u}_0 \text { and } x(0) := \hat{x}^N_0. \end{aligned}$$

and it is assumed that the following limit exists

12 $$\begin{aligned} \lim _{N\rightarrow \infty } \hat{x}^N_0&= \hat{x}_0. \end{aligned}$$

We assume the following uniform growth bounds: for some constant K>0, for all t≥0, and $u\in \mathbb {R}^m$, and all $\alpha \in \mathscr {R}$,

13 $$\begin{aligned} \left\| A( u , X ) \right\|&\le K \big (1 + \left\| u \right\| \big ) \end{aligned}$$

14 $$\begin{aligned} \big | \lambda _\alpha \big ( X , u \big ) \big |&\le K. \end{aligned}$$

Second, we assume that *A* and $\lambda _n$ are both uniformly Lipschitz in their arguments: that is,

15 $$\begin{aligned} \left\| A\big ( u, X \big ) -A\big ( \tilde{u}, \tilde{X} \big ) \right\|&\le C \big \lbrace \left\| u - \tilde{u} \right\| + \left\| X - \tilde{X} \right\| \big \rbrace \end{aligned}$$

16 $$\begin{aligned} \sup _{\alpha \in \mathscr {R}}\Vert \lambda _\alpha \big ( u, X \big ) -\lambda _\alpha \big ( \tilde{u}, \tilde{X} \big ) \Vert&\le C\big \lbrace \left\| u - \tilde{u} \right\| + \left\| X - \tilde{X} \right\| \big \rbrace . \end{aligned}$$

Standard ODE theory dictates that for any piecewise-continuous *x*, there is a unique solution to the ODE in Eq. (10). See Davis for more details on why this is a well-defined stochastic system (Davis 1984). In fact its easy to prove a unique solution to the time-rescaled representation in Eq. (17). One simply iterates between the jump-times of $Y_{\alpha }(t)$, solving the ODE (10) between these times.

The linearity of Poisson Processes ensures that $Z_{\alpha }(t)$ can be represented as, writing $\lbrace Y_{\alpha }(t) \rbrace _{\alpha \in \mathscr {R}}$ to be independent unit-intensity Poisson Processes (Anderson and Kurtz 2015),

17 $$\begin{aligned} Z_{\alpha }(t) =&Y_{\alpha }\bigg (N \int _0^t \lambda _{\alpha }\big ( x(s) , u(s) \big ) ds \bigg ) \end{aligned}$$

18 $$\begin{aligned} z_{\alpha }(t) =&N^{-1}Z_{\alpha }(t) \text { with initial condition} \end{aligned}$$

19 $$\begin{aligned} z_{\alpha }(0) =&0. \end{aligned}$$

Writing $y_{\alpha }(t) = N^{-1}Y_{\alpha }(Nt)$, it may be observed that

20 $$\begin{aligned} z_{\alpha }(t) = y_{\alpha }\bigg ( \int _0^t \lambda _{\alpha }\big ( x(s) , u(s) \big ) ds \bigg ). \end{aligned}$$

The main result of this paper is the following theorem. Further below in Eq. (24) we specify Υ to be the state space that the triple (*z*, *x*, *u*) lives in (and we define a topology too). The sequence of probability laws of $\big ( z,x,u \big )$ satisfies the following asymptotic estimate on the space Υ as $N\rightarrow \infty$.

### Theorem 1.1

Suppose that $\mathcal {O},\mathcal {A} \subset \Upsilon$, with $\mathcal {O}$ open and $\mathcal {A}$ closed, are such that for any T>0,

21 $$\begin{aligned} \inf _{ (z,x,u) \in \mathcal {A} \cup \mathcal {O}} \inf _{\alpha \in \mathscr {R}} \inf _{s\in [0,T]} \lambda _\alpha (x(s),u(s)) > 0. \end{aligned}$$

Then there exists a function $\mathcal {J}: \Upsilon \rightarrow \mathbb {R}$ (specified in Sect. 1.1) that is (i) lower-semi-continuous and (ii) has compact level sets, such that

22 $$\begin{aligned} \underset{N\rightarrow \infty }{\overline{\lim }}N^{-1}\log \mathbb {P}\big ( (z,x,u) \in \mathcal {A} \big )&\le - \inf _{(z,x,u) \in \mathcal {A}}\mathcal {J}(z,x,u) \end{aligned}$$

23 $$\begin{aligned} \underset{N\rightarrow \infty }{\underline{\lim }}N^{-1}\log \mathbb {P}\big ( (z,x,u) \in \mathcal {O} \big )&\ge - \inf _{(z,x,u)\in \mathcal {O}}\mathcal {J}(z,x,u). \end{aligned}$$

### Topological definitions

Before we state our main result we must briefly note the topological space that our variables belong to. Write the state space for $\big ( z,x, u \big )$ as Υ, i.e.

24 $$\begin{aligned} &  \Upsilon := \big \lbrace (z,x,u) \in \tilde{\mathcal {D}}([0,\infty ),\mathbb {R}^+)^{M} \times \mathcal {D}([0,\infty ),\mathbb {R}^+)^{d} \times \mathcal {C}^1_{piece} ([0,\infty ), \mathbb {R}^m) \nonumber \\ &  \text { where (i)}\sum _{\alpha \in \mathscr {R}} z_{\alpha }(t) \zeta _{\alpha } = x(t) -x(0),\; \text {(ii) for all }t\ge 0, \; \; \frac{du}{dt} \nonumber \\ &  = A\big ( u(t), x(t) \big ) \text { and }u(0) = \hat{u}\nonumber \\ &  \text { and }(iii)~ z(0) = 0 \big \rbrace \end{aligned}$$

and endow Υ with the product topology. Let $\Upsilon _* \subset \Upsilon$ consist of all (*z*, *x*, *u*) such that $x(0) = \hat{x}_0$. Since the values of *x* and *u* are effectively determined by *z*, we only need to metrize the convergence of *z*. This is captured by the following lemma. Recall that $\tilde{\mathcal {D}}([0,\infty ),\mathbb {R}^+)$ consists of all non decreasing paths that start at zero.

#### Lemma 1.2

Υ is a closed subset of $\tilde{\mathcal {D}}([0, \infty ), \mathbb {R}^+)^M \times \mathcal {D}([0, \infty ), \mathbb {R}^+)^d \times \mathcal {C}_{piece}^1([0, \infty ), \mathbb {R})^m$. Υ is separable and metrizable. Furthermore for any $(z, x, u) \in \Upsilon$, any t,δ>0, there exists $\tilde{\delta } > 0$ such that

25 $$\begin{aligned} \left\{ (\tilde{z},\tilde{x},\tilde{u}) \in \Upsilon : \sup _{\alpha \in \mathscr {R}} d_\infty ^\circ (z_\alpha , \tilde{z}_\alpha ) \le \tilde{\delta } \text { and } \Vert x(0) - \tilde{x}(0)\Vert \le \tilde{\delta } \right\} \subseteq \qquad \qquad \qquad \qquad \end{aligned}$$

26 $$\begin{aligned} \left\{ (\tilde{z},\tilde{x},\tilde{u}) \in \Upsilon \times \Upsilon : \sup _{\alpha \in \mathscr {R}} d_\infty ^\circ (z_\alpha , \tilde{z}_\alpha ) \le \delta \right. \qquad \qquad \qquad \qquad \qquad \end{aligned}$$

27 $$\begin{aligned} \left. \text {and } \sup _{j \le d} d_\infty ^\circ (x_j, \tilde{x}_j) \le \delta \text { and } \sup _{0 \le s \le t} \Vert u(s) - \tilde{u}(s)\Vert \le \delta \right\} \end{aligned}$$

We can now define the rate function

28 $$\begin{aligned} \mathcal {J}(z,x,u): \Upsilon \mapsto \mathbb {R}^+, \end{aligned}$$

starting by stipulating that it is infinite if any of the following conditions are satisfied (i) $z_{\alpha }: \mathbb {R}^+ \rightarrow \mathbb {R}^+$ is not absolutely continuous for any $\alpha \in \mathscr {R}$, (ii) $x(0) \ne \hat{x}_0$. Otherwise, if $z_{\alpha }$ is absolutely continuous for every $\alpha \in \mathscr {R}$ (which means that it must have a derivative $\dot{z}_{\alpha }$ for Lebesgue almost every time), we make the definition

29 $$\begin{aligned} \mathcal {J}\big ( z,x,u \big )&= \sum _{\alpha \in \mathscr {R}}\int _{\mathbb {R}^+ } \ell \bigg ( \dot{z}_{\alpha }(r) / \lambda _{\alpha }(x(r),u(r)) \bigg )\lambda _{\alpha }(x(r),u(r)) dr\text { where } \end{aligned}$$

30 $$\begin{aligned} \ell (a)&= a\log a -a +1. \end{aligned}$$

## Euler–Lagrange equations

In this section we determine the structure of the Euler–Lagrange equations that determine the most likely trajectory followed by the system in attaining a specified state. Computing estimates for first-hitting-times is one of the most important applications of large deviations theory (Newby 2014). For the application of large deviations theory to first-hitting-time estimates, see for example (Weinan and Vanden-Eijnden 2010; Grafke and Vanden-Eijnden 2017; MacLaurin and Newby 2025). The ‘fast processes’ (i.e. the ODE in *u*(*t*)) are implemented as a constraint (with Lagrange multipliers). We start by framing the problem in terms of the reaction fluxes; that is, we wish to determine the most likely trajectory followed by the system in attaining a target reaction flux $z^* \in (\mathbb {R}^+)^{|\mathscr {R}|}$ at time *T*. We must determine the trajectory

$$ (z,x,u) \in \mathcal {C}^1(\mathbb {R})^M \times \mathcal {C}^1(\mathbb {R})^d \times \mathcal {C}^1(\mathbb {R})^m $$

such that $\mathcal {J}_T$ is minimized, where

31 $$\begin{aligned} \mathcal {J}_T = \int _0^T L\big ( \dot{z}(s), x(s) , u(s) \big ) ds, \end{aligned}$$

where $L: \mathbb {R}^M \times \mathbb {R}^d\times \mathbb {R}^m \ \rightarrow \mathbb {R}^+$ is the integrand of the rate function in Eq. (29), i.e.

32 $$\begin{aligned} L\big (q,x , u \big )&= \sum _{\alpha \in \mathscr {R}} \ell \big ( q_{\alpha } / \lambda _{\alpha }(x,u) \big )\lambda _{\alpha }(x,u) \end{aligned}$$

33 $$\begin{aligned} \ell (a)&= a\log a -a +1. \end{aligned}$$

subject to the following constraints and boundary conditions

34 $$\begin{aligned} \frac{du}{dt} =&A(u,x) \end{aligned}$$

35 $$\begin{aligned} x(t) =&x(0) + \sum _{\alpha \in \mathscr {R}}z_{\alpha }(t) \xi _{\alpha } . \end{aligned}$$

36 $$\begin{aligned} u(0) =&\hat{u}\end{aligned}$$

37 $$\begin{aligned} z_{\alpha }(0) =&0 \end{aligned}$$

38 $$\begin{aligned} z_{\alpha }(T) =&z_{\alpha }^*. \end{aligned}$$

We introduce Lagrange Multipliers $\lbrace \eta ^i(t) \rbrace _{1\le i \le m}$ corresponding to the *m* constraints

39 $$\begin{aligned} \frac{du^i_t}{dt} = A^i(u_t, x_t). \end{aligned}$$

Our task is now to find the critical points of the functional

40 $$\begin{aligned}&\tilde{\mathcal {J}}_T( z,u,\eta ) = \int _0^T \tilde{L}\big ( \dot{z}(s), x(s) , u(s) \big ) ds \text { where } \end{aligned}$$

41 $$\begin{aligned}&\tilde{L}\big ( \dot{z}(s), x(s) , u(s) \big ) = L\big ( \dot{z}(s), x(s) , u(s) \big ) \nonumber \\&-\sum _{i=1}^m \eta ^i(s) \big \lbrace \dot{u}^i(s)- A^i(u(s),x(s)) \big \rbrace \text { such that }\end{aligned}$$

42 $$\begin{aligned}&x(t) = \hat{x}(0) + \sum _{\alpha \in \mathscr {R}}z_{\alpha }(t) \xi _{\alpha } . \end{aligned}$$

We now take the Fréchet Derivative of (40), i.e. for $w \in \mathcal {C}^1([0,T],\mathbb {R})^M$ and $v \in \mathcal {C}^1([0,T],\mathbb {R})^m$, and $\kappa \in \mathcal {C}^1([0,T],\mathbb {R})^m$, with boundary conditions w(0)=0,w(T)=0,v(0)=0, and the boundary conditions for κ are not yet clear. Thanks to the constraint in Eq. (42), we have that

43 $$\begin{aligned} \frac{d}{dz_{\alpha }} = \sum _{i=1}^d \frac{\partial }{\partial x^i} \xi ^i_{\alpha }. \end{aligned}$$

We therefore find that

$$\begin{aligned}\begin{array}{c} D \tilde{\mathcal {J}}_T( z,u,\eta ) \cdot (w,v,\kappa ):= \lim _{\epsilon \rightarrow 0^+}\epsilon ^{-1} \big \lbrace \tilde{\mathcal {J}}_T( z + \epsilon w,u + \epsilon v,\eta + \epsilon \kappa ) - \tilde{\mathcal {J}}_T( z,u,\eta ) \big \rbrace \\ =\sum _{\alpha \in \mathscr {R}} \int _0^T w_{\alpha }(t) \bigg \lbrace \sum _{i=1}^d \bigg ( \frac{\partial L}{\partial x^i}\xi ^i_{\alpha } + \sum _{k=1}^m \eta ^k \frac{\partial A^k}{\partial x^i} \xi ^i_{\alpha } \bigg ) - \frac{d}{dt} \frac{\partial L}{\partial \dot{z}_{\alpha }} \bigg \rbrace dt \nonumber \\ + \sum _{i=1}^m\int _0^T v^i(t) \bigg \lbrace \frac{\partial L}{\partial u^i} + \frac{d\eta ^i}{dt} + \sum _{k=1}^m \eta ^k \frac{\partial A^k}{\partial u^i} \bigg \rbrace \\ -\kappa ^i \bigg \lbrace \frac{du^i}{dt} - A^i(u(t),x(t)) \bigg \rbrace dt\\ + \sum _{\alpha \in \mathscr {R}}\bigg \lbrace w_{\alpha }(T)\frac{\partial }{\partial \dot{z}_{\alpha }}L(\dot{z}(T),x(T), u(T)) - w_{\alpha }(0)\frac{\partial }{\partial \dot{z}_{\alpha }}L(\dot{z}(0),x(0), u(0))\bigg \rbrace \\ - \sum _{i=1}^m \big \lbrace \eta ^i(T) v^i(T) - \eta ^i(0) v^i(0) \big \rbrace , \end{array}\end{aligned}$$

and to obtain the above expression, we have used integration by parts to write

44 $$\begin{aligned} &  \sum _{\alpha \in \mathscr {R}} \int _0^T \dot{w}_{\alpha }(t) \frac{\partial L}{\partial \dot{z}_{\alpha }} dt = \sum _{\alpha \in \mathscr {R}}\bigg \lbrace w_{\alpha }(T)\frac{\partial }{\partial \dot{z}_{\alpha }}L(\dot{z}(T),x(T), u(T)) \nonumber \\ &  - w_{\alpha }(0)\frac{\partial }{\partial \dot{z}_{\alpha }}L(\dot{z}(0),x(0), u(0)) \nonumber \\ &  - \int _0^T w_{\alpha }(t)\frac{d}{dt} \frac{\partial L}{\partial \dot{z}_{\alpha }} dt \bigg \rbrace \end{aligned}$$

and

45 $$\begin{aligned} \sum _{i=1}^m \int _0^t \eta ^i(t) \dot{v}^i(t) dt = \sum _{i=1}^m \big \lbrace \eta ^i(T) v^i(T) - \eta ^i(0) v^i(0) -\int _0^t \dot{\eta }^i(t) v^i(t) dt \big \rbrace . \end{aligned}$$

Setting the coefficients of $w_{\alpha }$, *v* and $\kappa ^i$ to zero, we obtain the equations

46 $$\begin{aligned} \sum _{i=1}^d \bigg (\frac{\partial L}{\partial x^i}\xi ^i_{\alpha } + \sum _{k=1}^m \eta ^k \frac{\partial A^k}{\partial x^i} \xi ^i_{\alpha } \bigg ) - \frac{d}{dt} \frac{\partial L}{\partial \dot{z}_{\alpha }}&= 0 \end{aligned}$$

47 $$\begin{aligned} \frac{\partial L}{\partial u^i}+ \frac{d\eta ^i}{dt} + \sum _{k=1}^m \eta ^k \frac{\partial A^k}{\partial u^i}&= 0 \end{aligned}$$

48 $$\begin{aligned} \frac{du^i}{dt} - A^i(u(s),x(s) )&= 0 \end{aligned}$$

49 $$\begin{aligned} \eta ^i(T)&= 0. \end{aligned}$$

Noting that $\dot{\ell }(a) = \log a$, we compute that $\frac{\partial L}{\partial \dot{z}_{\alpha }} = \log \frac{\dot{z}_{\alpha }}{\lambda _{\alpha }(x(t),u(t))}$, and therefore

50 $$\begin{aligned} \frac{d}{dt} \frac{\partial L}{\partial \dot{z}_{\alpha }} =&\frac{d}{dt}\left( \log \frac{\dot{z}_{\alpha }}{\lambda _{\alpha }(x(t),u(t))} \right) \end{aligned}$$

51 $$\begin{aligned} =&\frac{\ddot{z}_{\alpha }}{\dot{z}_{\alpha }} - \frac{1}{\lambda _{\alpha }(x(t),u(t))} \frac{d}{dt}\lambda _{\alpha }(x(t),u(t)). \end{aligned}$$

Thus in the case that $\lambda _{\alpha }(x,u) > 0$, the Euler Lagrange equations are such that for $1\le j \le m$, $\alpha \in \mathscr {R}$ and $1\le i \le d$,

52 $$\begin{aligned} \frac{\ddot{z}_{\alpha }}{\dot{z}_{\alpha }} =&\frac{1}{\lambda _{\alpha }} \frac{d\lambda _{\alpha }}{dt}+\sum _{i=1}^d \xi ^i_{\alpha } \frac{\partial L}{\partial x_i} - \sum _{k=1}^d \sum _{j=1}^m \eta ^j(t) \frac{\partial A^j}{\partial x^k_t} \xi ^k_{\alpha } \end{aligned}$$

53 $$\begin{aligned} \frac{d\eta ^j}{dt} =&- \frac{\partial L}{\partial u^j} - \sum _{k=1}^m \eta ^k \frac{\partial A^k}{\partial u^j} \end{aligned}$$

54 $$\begin{aligned} x^i(t) =&x^i(0) + \sum _{\alpha \in \mathscr {R}}z_{\alpha }(t) \xi ^i_{\alpha } \text { for all }t\ge 0 . \end{aligned}$$

55 $$\begin{aligned} \eta ^j (T) =&0 \end{aligned}$$

56 $$\begin{aligned} z_{\alpha }(0) =&0 \end{aligned}$$

57 $$\begin{aligned} z(T) =&z_*. \end{aligned}$$

For the sake of completeness, we note that

58 $$\begin{aligned} \frac{d\lambda _{\alpha }}{dt} =&\sum _{\beta \in \mathscr {R}} \sum _{i=1}^d \frac{\partial \lambda _{\alpha }}{\partial x^i}\xi ^i_{\beta } \dot{z}_{\beta }+\sum _{i=1}^m \frac{\partial \lambda _{\alpha }}{\partial u^i}A^i(u(t),x(t)) \end{aligned}$$

59 $$\begin{aligned} \frac{\partial L}{\partial u^i} =&\sum _{\alpha \in \mathscr {R}}\frac{\partial \lambda _{\alpha }}{\partial u^i} \bigg (1 - \frac{\dot{z}_{\alpha }}{\lambda _{\alpha }} \bigg ) \end{aligned}$$

It can be seen that this system amounts to an ODE boundary value problem. One way that one may attempt to solve this problem is via the shooting method (Weinan and Vanden-Eijnden 2010). At time 0, the unknowns are $\lbrace \dot{z}_{\alpha }(0) \rbrace _{\alpha \in \mathscr {R}}$ and $\lbrace \eta ^j(0) \rbrace _{1\le j \le m}$. The shooting method requires one to guess values for the unknowns at time 0, (ii) integrate forward the equations to time *T*, and then (iii) implement the constraints $\lbrace \dot{z}_{\alpha }(T) = \dot{z}_{*,\alpha } \rbrace _{\alpha \in \mathscr {R}}$ and $\lbrace \eta ^j(T) = 0 \rbrace _{1\le j \le m}$. Another numerical method, which can be much more numerically efficient, is the geometric method of Vanden-Eijnden and co-workers (Heymann and Vanden-Eijnden 2008; Grafke et al. 2024).

### Simplified Euler–Lagrange equations

Usually, one desires to know the first-hitting-time for the concentrations $\lbrace x^i(T) \rbrace _{1\le i \le d}$, rather than the first hitting time for the reaction fluxes. That is, for a fixed $x_* \in \mathbb {R}^d$, one wishes to estimate the probability that $x_T \simeq x_*$, and also determine the optimal (most likely) path followed by the system in attaining this point. For this reason, it can be simpler to eliminate the reaction fluxes and reduce the problem to one purely in terms of the concentrations and the ODE variables $\lbrace u^i(T) \rbrace _{1\le i \le m}$. To this end, for any $\dot{x} \in \mathbb {R}^d$, $x \in (\mathbb {R}^+)^d$, and $u \in \mathbb {R}^m$, define the set

60 $$\begin{aligned} \Xi (\dot{x},x,u) = \big \lbrace \dot{z} \; : \; \dot{x}^p =\sum _{\alpha \in \mathscr {R}} \xi ^p_{\alpha } \dot{z}_{\alpha } \big \rbrace \subset (\mathbb {R}^+)^M \end{aligned}$$

Then define the function

61 $$\begin{aligned} \hat{L}\big ( \dot{x}(s), x(s) , u(s) \big ) = \inf \big \lbrace L( \dot{z}, x(s) , u(s) ) \; : \; \dot{z} \in \Xi (\dot{x},x,u) \big \rbrace \end{aligned}$$

In the case that $\Xi (\dot{x},x,u) = \emptyset$, define $\hat{L}\big ( \dot{x}(s), x(s), u(s) \big ) = \infty$. One can thus define the contracted rate function

62 $$\begin{aligned} \tilde{\mathcal {J}}_T:&\mathcal {C}_{ac}([0,T],(\mathbb {R}^+)^d \big ) \times \mathcal {C}_{ac}([0,T],\mathbb {R}^m \big ) \rightarrow \mathbb {R} \end{aligned}$$

63 $$\begin{aligned} \tilde{\mathcal {J}}_T(x,u)&= \int _0^T \hat{L}\big ( \dot{x}(s), x(s) , u(s) \big ) ds, \end{aligned}$$

and it is immediate from the Contraction Principle (Dembo and Zeitouni 1998) that $\tilde{\mathcal {J}}_T$ governs the large deviations of (*x*, *u*). The following convexity property makes it easier to compute optimal trajectories.

#### Lemma 2.1

$\hat{L}$ is convex in its first argument.

#### Proof

This is because Ξ is linear in $\dot{x}$, and *L* is convex in its first argument. □

Next, we outline the Euler–Lagrange equations for $\tilde{\mathcal {J}}_T$. The derivation parallels that of the previous section. Our task is to find the critical points of the functional

64 $$\begin{aligned} \grave{\mathcal {J}}_T( x,u,\eta ) =&\int _0^T \grave{L}\big ( \dot{x}(s), x(s) , u(s) \big ) ds \text { where } \end{aligned}$$

65 $$\begin{aligned} \grave{L}\big ( \dot{x}(s), x(s) , u(s) , \eta (s) \big ) =&\hat{L}\big ( \dot{x}(s), x(s) , u(s) \big ) -\sum _{i=1}^m \eta ^i(s) \big \lbrace \dot{u}^i(s)- A^i(u(s),x(s)) \big \rbrace , \end{aligned}$$

and $\eta ^i \in \mathcal {C}^1([0,T],\mathbb {R})$ is the Lagrange Multiplier.

We now take the Frechet Derivative of (64), i.e. for $w \in \mathcal {C}^1([0,T],\mathbb {R})^M$ and $v \in \mathcal {C}^1([0,T],\mathbb {R})^m$, and $\kappa \in \mathcal {C}^1([0,T],\mathbb {R})^m$, with boundary conditions w(0)=0,w(T)=0,v(0)=0, and the boundary conditions for κ are not yet clear,

$$\begin{aligned} &  D \grave{\mathcal {J}}_T( x,u,\eta ) \cdot (w,v,\kappa ):= \lim _{\epsilon \rightarrow 0^+}\epsilon ^{-1} \big \lbrace \grave{\mathcal {J}}_T( x + \epsilon w,u + \epsilon v,\eta + \epsilon \kappa ) - \tilde{\mathcal {J}}_T( x,u,\eta ) \big \rbrace \\ &  \quad = \int _0^T \sum _{i=1}^d w_{i}(t) \bigg \lbrace \bigg ( \frac{\partial \hat{L}}{\partial x^i} +\sum _{k=1}^m \eta ^k \frac{\partial A^k}{\partial x^i} \bigg ) - \frac{d}{dt} \frac{\partial \hat{L}}{\partial \dot{x}^i} \bigg \rbrace dt \nonumber \\ &  \quad + \sum _{i=1}^m\int _0^T v^i(t) \bigg \lbrace \frac{\partial \hat{L}}{\partial u^i} + \frac{d\eta ^i}{dt} + \sum _{k=1}^m \eta ^k \frac{\partial A^k}{\partial u^i} \bigg \rbrace \\ &  \quad c-\kappa ^i \bigg \lbrace \frac{du^i}{dt} - A^i(u(t),x(t)) \bigg \rbrace dt\\ &  - \sum _{i=1}^m \big \lbrace \eta ^i(T) v^i(T) - \eta ^i(0) v^i(0) \big \rbrace \end{aligned}$$

The Euler–Lagrange equations are thus, for $1\le i \le d$ and $1\le j \le m$,

66 $$\begin{aligned} \frac{\partial \hat{L}}{\partial x^i} + \sum _{k=1}^m \eta ^k \frac{\partial A^k}{\partial x^i} - \frac{d}{dt} \frac{\partial \hat{L}}{\partial \dot{x}^i}&= 0 \end{aligned}$$

67 $$\begin{aligned} \frac{\partial \hat{L}}{\partial u^j}+ \frac{d\eta ^j}{dt} + \sum _{k=1}^m \eta ^k \frac{\partial A^k}{\partial u^j}&= 0 \end{aligned}$$

68 $$\begin{aligned} \frac{du^i}{dt} - A^i(u(s),x(s) )&= 0 \end{aligned}$$

69 $$\begin{aligned} \eta ^i(T)&= 0 . \end{aligned}$$

For the sake of completeness, we note that the derivative assumes the form

70 $$\begin{aligned} \frac{d}{dt} \frac{\partial \hat{L}}{\partial \dot{x}^i} =\sum _{j=1}^d \bigg \lbrace \frac{\partial ^2 \hat{L}}{\partial \dot{x}^i \partial \dot{x}^j} \ddot{x}^j + \frac{\partial ^2 \hat{L}}{\partial \dot{x}^i \partial x^j} \dot{x}^j \bigg \rbrace +\sum _{j=1}^m \frac{\partial ^2 \hat{L}}{\partial \dot{x}^i \partial u^j} \frac{du^j}{dt} \end{aligned}$$

## Application to calcium signalling

We apply this theory to determine estimates for the typical time it takes for cells with stochastically opening and closing calcium channels to change from most of the channels being closed to most being open (Dupont et al. 2016). As noted in the Introduction, it is widely conjectured that intracellular calcium waves are essentially stochastic phenomena (Keizer and Smith 1998; Keener 2006; Rüdiger 2014; Dupont et al. 2016; Ramlow, Falcke and Lindner 2023b). The waves do not always start from the same ion channel in the cell. Rather rare stochastic fluctuations of a channel can sometimes lead to a ‘puff’ of calcium in a cluster of channels, which sometimes leads to a wave spreading throughout the cell. Thus scholars such as Keizer and Smith (1998) and Ramlow, Falcke and Lindner (2023b) hypothesize that a ‘hierarchical’ explanation is needed, i.e. one must explain how microscopic stochasticity at the level of ion channels is organized to produce coherent cell-wide waves.

There are two components to our model of calcium dynamics: the opening and closing channels, and the calcium concentration (that evolves deterministically between the opening and closing of the channels). The model is simplified to be such that the calcium concentration throughout the cell is spatially homogeneous. Following for instance (Coombes and Timofeeva 2003; Keener 2006; Ramlow et al. 2023a; Ramlow, Falcke and Lindner 2023b), we employ a hybrid model: with the calcium diffusion modeled deterministically, the IP3 concentration constant throughout the cell, and the channel opening and closing modeled stochastically.

Our basic aim is to determine the probability of calcium puffs and waves by studying a similar stochastic model to Coombes and Timofeeva (2003); Keener (2006); Ramlow et al. (2023a); Ramlow, Falcke and Lindner (2023b). These papers performed detailed numerical simulations, and analytically estimated the probabilities. To calculate the probability of a calcium puff in a cluster, the calcium and IP3 concentration throughout the cluster is assumed to be homogeneous. However there is a feedback effect of the opening / closing of the channels: when these bind or unbind the calcium / IP3, they alter the local concentration. Thus the opening and closing of channels within a cluster is not independent because the channels communicate with each other via the calcium / IP3 concentrations.

### Piecewise-deterministic-Markov-process model of calcium dynamics

We suppose that there are *N* channels distributed on the cell membrane. It is assumed that spatial effects are negligible, so that the calcium concentrations can be modelled as approximately spatially homogeneous using ODEs. Let $u_1$ be the calcium concentration in the cellular cytosol, and $u_2$ be the calcium concentration in the endoplasmic reticulum.

We take the number of stochastic variables corresponding to each channel to be 1. That is, $q^i_t = 1$ if the channel is open, and $q^i_t = 0$ if the channel is closed. Employing a standard model (Dupont et al. 2016) for the calcium dynamics, we obtain that

71 $$\begin{aligned} \frac{du_1}{dt} =&k_f x(t) (u_2 - u_1) - J_{serca}(u) + J_{leak} := A^1(u,x) \end{aligned}$$

72 $$\begin{aligned} \frac{du_2}{dt} =&\gamma J_{serca}(u_1) - \gamma k_f x(t) (u_2 - u_1) - \gamma J_{leak} := A^2(u,x), \end{aligned}$$

73 $$\begin{aligned} J_{serca}(u_1) =&\frac{V_s (u_1)^2}{K_s^2 + (u_1)^2} \end{aligned}$$

74 $$\begin{aligned} J_{leak}(u) =&k_{leak}(u_2 - u_1) \end{aligned}$$

One may observe that

75 $$\begin{aligned} \frac{d}{dt}\big ( \gamma u_1 + u_2 \big ) = 0, \end{aligned}$$

which means that we can eliminate $u_2$ from the dynamics, and write $u:= u_1(t)$, and $A^1:= A$, where

76 $$\begin{aligned} A(u,x) = (\gamma -1) k_f x u - J_{serca}(u) + J_{leak} . \end{aligned}$$

Now

77 $$\begin{aligned} x(t) = \frac{1}{N}\sum _{j=1}^N q^j_t. \end{aligned}$$

The Markovian switching of the channel is given by

- Reaction 1 (Closing of a Channel): $q^j_t$ switches from 1 to 0 with intensity $\lambda _1(u,x) = \alpha _{-1}x$. The stoichiometric constant is $\zeta _1 = -1$.
- Reaction 2 (Opening of a Channel): $q^j_t$ switches from 0 to 1 with intensity $\lambda _2(u,x) = \alpha _1 u_1 (1-x)$. The stoichiometric constant is $\zeta _2 = 1$.

We note that our proof of the large deviation principle required the reaction rates to be bounded away from 0. We can thus only apply the LDP to estimate rare events for x∈(0,1). Let $z_1(t)$ count the number of times reaction 1 happens (and divided by *N*), and $z_2(t)$ count the number of times that reaction 2 happens (and divided by *N*). Then it must be that

78 $$\begin{aligned} x(t) = x(0) + z_2(t) - z_1(t). \end{aligned}$$

#### Large *N* limiting dynamics.

As $N\rightarrow \infty$ it is classical that the concentration of open channels converges to the following ODE (Kurtz 1971; Ethier and Kurtz 1986).

79 $$\begin{aligned} \frac{dx}{dt} =&\lambda _2(u,x) - \lambda _1(u,x). \end{aligned}$$

80 $$\begin{aligned} \frac{du_1}{dt} =&k_f x(t) (u_2 - u_1) - J_{serca}(u) + J_{leak} := A^1(u,x) \end{aligned}$$

81 $$\begin{aligned} \frac{du_2}{dt} =&\gamma J_{serca}(u_1) - \gamma k_f x(t) (u_2 - u_1) - \gamma J_{leak} := A^2(u,x), \end{aligned}$$

For the parameters that we choose, there exists a unique stable fixed point of (79) - (81). This point is written as $(x^*, u_1^*, u_2^*)$. These equations are excitable (in the mathematical sense) (Lindner et al. 2004; Dupont et al. 2016). One therefore expects that a sufficient amount of noise can excite a large response in the system (Lindner et al. 2004). Mathematically, the mechanism for producing calcium waves should be broadly analogous to the stochastic Morris-Lecar model explored by Newby (2014).

### Optimal trajectories

We wish to predict the probability of the proportion of open calcium channels being significantly greater than its equilibrium value at some time *T*. *T* is taken to be of the order of the experimentally-observed timescale of calcium puffs. We write the ‘target’ proportion of channels to be $\hat{x} \in (x_*, 1)$. We thus wish to compute the trajectory $(x,u) \in \mathcal {A} \subset \mathcal {C}^2([0,T],\mathbb {R}) \times \mathcal {C}^1([0,T],\mathbb {R}^2)$ such that

82 $$\begin{aligned} \tilde{\mathcal {J}}_T(x,u) = \inf \big \lbrace \tilde{\mathcal {J}}_T(y,v) \; : \; (y,v) \in \mathcal {A} \big \rbrace \end{aligned}$$

and $\mathcal {A} \subset \mathcal {C}^2([0,T],\mathbb {R}) \times \mathcal {C}^1([0,T],\mathbb {R}^2)$ consists of all (*x*, *u*) such that $x(0) = x^*$,

83 $$\begin{aligned} x(T) = \hat{x}, \end{aligned}$$

$u(0) = u^*$ and for all t∈[0,T],

84 $$\begin{aligned} \frac{du}{dt}= A(u(t),x(t) ). \end{aligned}$$

The trajectory that minimizes $\tilde{\mathcal {J}}_T$ indicates the most likely means that a significant proportion of calcium channels open over the time interval [0, *T*] due to stochastic effects. In the next section we employ Calculus of Variations to compute the optimal trajectory.

#### Euler–Lagrange equations

We work with the contracted rate function in Eq. (61). We first find a much simpler form for the contracted rate function. Because our LDP is only valid for non-vanishing reaction rates, we restrict *x* to lie between δ and 1-δ for some $\delta \in (0,1)$.

##### Lemma 3.1

For $$\delta< x < 1 - \delta $$,

85 $$\begin{aligned} \hat{L}\big ( \dot{x}, x , u \big ) = \ell \bigg ( \frac{\dot{z}_1}{\lambda _1(x,u)} \bigg )\lambda _1(x,u) + \ell \bigg ( \frac{\dot{x} + \dot{z}_1}{\lambda _2(x,u)} \bigg )\lambda _2(x,u) \end{aligned}$$

where $\dot{z}_1$ is such that

86 $$\begin{aligned} \dot{z}_1 \big ( \dot{x} + \dot{z}_1 \big ) = \lambda _1(x,u) \lambda _2(x,u). \end{aligned}$$

One must make sure to choose the root of the above quadratic that is such that $\dot{z}_1 \ge 0$.

##### Proof

It must be that

$$ \dot{x} = \dot{z}_2 - \dot{z}_1. $$

Thus, upon substitution, the Lagrangian assumes the form in (85). We must choose $\dot{z}_1$ to minimize (85), subject to the constraint $0\le \dot{z}_1 \le \dot{x}$. Differentiating (85) with respect to $\dot{z}_1$, and setting the derivative to zero, we obtain that

87 $$\begin{aligned} \log \bigg ( \frac{\dot{z}_1}{\lambda _1(x,u)} \bigg ) + \log \bigg ( \frac{ \dot{z}_1 + \dot{x}}{\lambda _2(x,u)} \bigg ) = 0. \end{aligned}$$

Exponentiating both sides, we obtain (86). □

Adapted to the calcium-signalling model of this section, the original Euler–Lagrange equations in Eqs. (66)–(69) are thus,

88 $$\begin{aligned} \frac{\partial \hat{L}}{\partial x} + \frac{\partial A}{\partial x}&= \frac{d}{dt} \frac{\partial \hat{L}}{\partial \dot{x}} \end{aligned}$$

89 $$\begin{aligned} \frac{\partial \hat{L}}{\partial u}+ \frac{d\eta }{dt} + \eta \frac{\partial A}{\partial u}&= 0 \end{aligned}$$

90 $$\begin{aligned} \frac{du}{dt} - A(u(t),x(t) )&= 0 \end{aligned}$$

91 $$\begin{aligned} \eta (T)&= 0. \end{aligned}$$

Now

92 $$\begin{aligned} \frac{d}{dt} \frac{\partial \hat{L}}{\partial \dot{x}} =\frac{\partial ^2 \hat{L}}{\partial \dot{x}^2} \ddot{x} + \frac{\partial ^2 \hat{L}}{\partial \dot{x} \partial x} \dot{x}+\sum _{j=1}^2 \frac{\partial ^2 \hat{L}}{\partial \dot{x} \partial u_j} \frac{du_j}{dt} \end{aligned}$$

We thus find that the (constrained) Euler Lagrange equations are (i.e. the ODEs in (x,u,η)) are such that

93 $$\begin{aligned} \frac{\partial ^2 \hat{L}}{\partial \dot{x}^2} \ddot{x} =&- \frac{\partial ^2 \hat{L}}{\partial \dot{x} \partial x} \dot{x}- \frac{\partial ^2 \hat{L}}{\partial \dot{x} \partial u} \frac{du}{dt} + \frac{\partial \hat{L}}{\partial x} + \eta \frac{\partial A}{\partial x} \end{aligned}$$

94 $$\begin{aligned} \frac{d\eta }{dt} =&- \frac{\partial \hat{L}}{\partial u} - \eta \frac{\partial A}{\partial u} \end{aligned}$$

95 $$\begin{aligned} \frac{du}{dt} =&A(u(t),x(t) ) \end{aligned}$$

The boundary conditions are η(T)=0, $u(0) = u_*$, $x(0) = x_*$ and $x(T) =\hat{x}$ (the target concentration of open channels). The equations (93)–(95) amount to a system of second-order boundary value ODEs (noting that the derivatives of $\hat{L}$ and *A* have analytic expressions, outlined further below). They can be solved using the shooting method. To implement the shooting method, we must shoot for the unknowns at time 0 (i.e. $\dot{u}(0)$ and η(0)) by implementing the constraints at time *T* (i.e. $x(T) = \hat{x}$ and η(T)=0).

The partial derivatives of $\hat{L}$ can be obtained by differentiating (85). They are as follows. Letting $\dot{z}_1 \ge 0$ satisfy the implicit relationship in Eq. (86) (there is a unique solution for 0<x<1 and u>0), it holds that

96 $$\begin{aligned} \frac{\partial \hat{L}}{\partial \dot{x}}&= \log \bigg ( \frac{\dot{z}_1}{\lambda _1(x,u)} \bigg ) \frac{\partial \dot{z}_1}{\partial \dot{x}} + \log \bigg ( \frac{\dot{x} + \dot{z}_1}{\lambda _2(x,u)} \bigg )\bigg (1+ \frac{\partial \dot{z}_1}{\partial \dot{x}} \bigg ) \text { where }\end{aligned}$$

97 $$\begin{aligned} \frac{\partial \dot{z}_1}{\partial \dot{x}}&=- \frac{\dot{z}_1}{2\dot{z}_1 + \dot{x}} . \end{aligned}$$

The second derivatives are such that

98 $$\begin{aligned} \frac{\partial ^2 \dot{z}_1}{\partial \dot{x}^2}&= \frac{\dot{z}_1}{(2\dot{z}_1 + \dot{x})^2} \bigg ( 1 + \frac{\dot{x}}{2\dot{z}_1 + \dot{x}} \bigg ) \end{aligned}$$

99 $$\begin{aligned} \frac{\partial ^2 \hat{L}}{\partial \dot{x}^2}&= 2 \frac{\partial ^2 \dot{z}_1}{\partial \dot{x}^2}\log \bigg ( \frac{\dot{z}_1 (\dot{x} + \dot{z}_1)}{\lambda _1(x,u) \lambda _2(x,u)} \bigg ) + \frac{\lambda _1(x,u)}{\dot{x} + \dot{z}_1} \bigg ( 1 + \frac{\partial \dot{z}_1}{\partial \dot{x}} \bigg ) \end{aligned}$$

100 $$\begin{aligned} \frac{\partial ^2 \hat{L}}{\partial \dot{x} \partial x}&= - \frac{\partial \lambda _1(x,u)}{\partial x} \frac{1}{\lambda _1(x,u)}\frac{\partial \dot{z}_1}{\partial \dot{x}} - \frac{\partial \lambda _2(x,u)}{\partial x} \frac{1}{\lambda _2(x,u)}\bigg (1+ \frac{\partial \dot{z}_1}{\partial \dot{x}} \bigg ) \end{aligned}$$

101 $$\begin{aligned} \frac{\partial ^2 \hat{L}}{\partial \dot{x} \partial u}&= - \frac{\partial \lambda _1(x,u)}{\partial u} \frac{1}{\lambda _1(x,u)}\frac{\partial \dot{z}_1}{\partial \dot{x}} - \frac{\partial \lambda _2(x,u)}{\partial u} \frac{1}{\lambda _2(x,u)}\bigg (1+ \frac{\partial \dot{z}_1}{\partial \dot{x}} \bigg ) \end{aligned}$$

### Numerical implementation

The computations were carried out in MATLAB using the unreduced formulation of the calcium-channel model. The deterministic equilibrium

$$ (x^*,u_1^*,u_2^*) $$

was first computed from the stationarity conditions together with the conserved quantity

$$ \gamma u_1 + u_2 = c_{\textrm{total}}, $$

and this equilibrium was used as the initial state for both the deterministic and stochastic evolutions. In particular, the equilibrium computation is performed by reducing to a scalar equation for $u_1$, then reconstructing $x^*$ and $u_2^*$ from the constitutive relations of the model. Note that $0< x^{*} < 1$, which means that our large deviations theory applies.

For the stochastic system, the code simulates the piecewise-deterministic Markov process directly in continuous time. Between channel-jump events, the variables $(u_1,u_2)$ evolve according to the deterministic calcium ODEs with the fraction of open channels held fixed. The next jump time is generated from the integrated total hazard, and at that jump time one samples either an opening or closing event according to the instantaneous rates. Repeating this procedure produces sample paths of

$$ (x(t),u_1(t),u_2(t)), $$

from which the code estimates both the final-time rare-event probability

$$ \mathbb {P}\bigl (x(T)\ge \hat{x}\bigr ) $$

and the hitting probability

$$ \mathbb {P}\Bigl (\max _{0\le t\le T} x(t)\ge \hat{x}\Bigr ). $$

A preliminary scan over a grid of values of $(T,\hat{x})$ is used to select a target pair for which the final-time event is rare but still numerically observable with Monte Carlo.

The optimal path is then computed from the unreduced Hamiltonian boundary-value problem for the variables

$$ (x,u_1,u_2,p,\eta _1,\eta _2), $$

where *p* is the conjugate momentum associated with *x*, and $\eta _1,\eta _2$ are the Lagrange multipliers associated with the two calcium constraints. Thus the numerical problem is to solve a first-order two-point boundary-value system consisting of the state equations for $(x,u_1,u_2)$ together with the adjoint equations for $(p,\eta _1,\eta _2)$, subject to the boundary conditions

$$ x(0)=x^*,\qquad u_1(0)=u_1^*,\qquad u_2(0)=u_2^*, $$

$$ x(T)=\hat{x},\qquad \eta _1(T)=0,\qquad \eta _2(T)=0. $$

From the resulting solution, the action

$$ S^*(T,\hat{x}) $$

is computed by numerical quadrature of the corresponding Lagrangian density along the optimal trajectory. The code also monitors the boundary residual and the drift of the extended Hamiltonian as diagnostics of numerical accuracy.

Since the stochastic variable *x*(*T*) takes values on the lattice $\{0,1/N,\dots ,1\}$ whereas the optimal-control problem imposes the continuous endpoint condition $x(T)=\hat{x}$, the Monte Carlo comparison is performed by conditioning the stored PDMP trajectories on ending near the lattice point closest to $\hat{x}$. The code then computes the conditioned mean path and pointwise 10–90% bands for $(x,u_1,u_2)$, and compares these with the optimal trajectory. The large-deviation prediction is assessed primarily through the comparison

$$ S^*(T,\hat{x}) \qquad \text {versus}\qquad -\frac{1}{N}\log P_{\textrm{MC}}, $$

since the subexponential prefactor in front of $\exp (-N S^*)$ is not included in the asymptotic approximation.

**Fig. 1:**
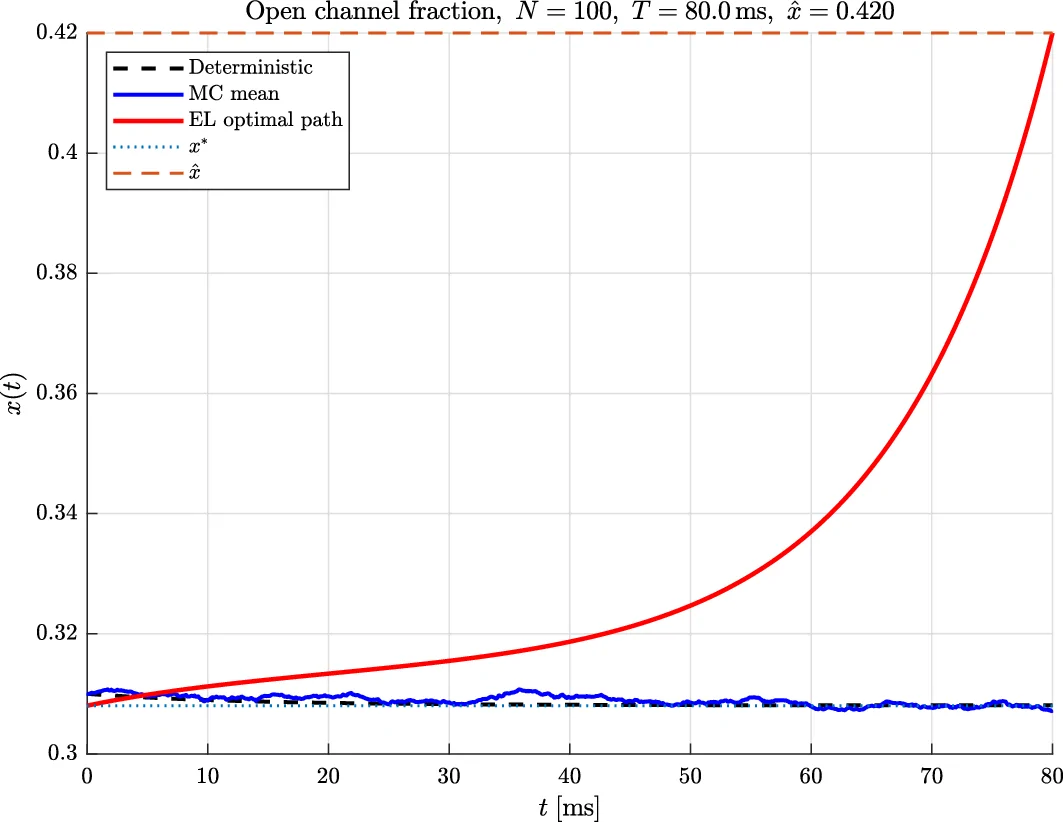
Comparison of the deterministic trajectory, the Monte Carlo mean trajectory, and the optimal Euler–Lagrange trajectory for the fraction of open channels *x*(*t*). The horizontal reference lines indicate the equilibrium value $x^*$ and the target value $\hat{x}$. This figure shows the rare excursion of the channel fraction from equilibrium toward the prescribed endpoint at time *T*

### Results

The parameters were taken from the textbook by Dupont et al. (2016):

**Figure:**
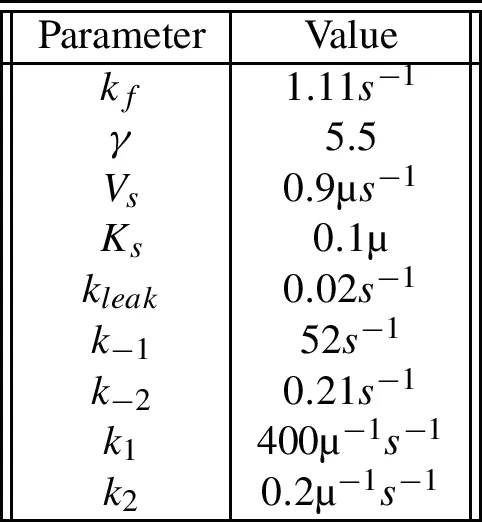

*Parameter selection.* To choose the pair $(T,\hat{x})$ we first perform a pilot Monte Carlo scan over

$$ T\in \{0.04,0.06,0.08\}\ \text {s}, \qquad \hat{x}\in \{0.42,0.46,0.48,0.52,0.56\}, $$

and estimate, for each pair, the final-time rare-event probability

$$ p_{\textrm{final}}=\mathbb {P}\bigl (x(T)\ge \hat{x}\bigr ). $$

The selected pair is chosen so that $p_{\textrm{final}}$ is close to a target value of order $10^{-2}$, while remaining inside a prescribed feasible window. In this way, the event is rare enough for the large-deviation approximation to be meaningful, but not so rare that Monte Carlo estimates become statistically unstable or that too few conditioned rare trajectories are available for comparison with the optimal path. In particular, choosing an event of probability of order $10^{-2}$ yields a sample of rare realizations large enough to estimate both $\mathbb {P}(x(T)\ge \hat{x})$ and the conditioned rare-path statistics with reasonable accuracy.

**Fig. 2:**
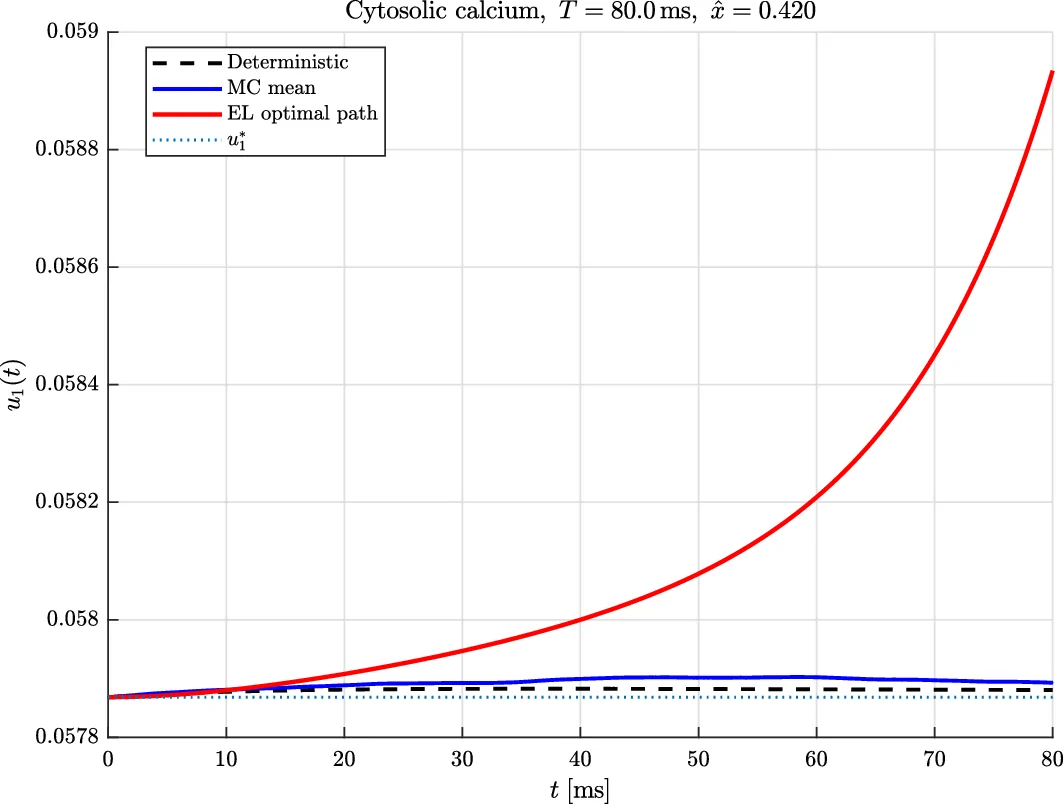
Comparison of the deterministic trajectory, the Monte Carlo mean trajectory, and the optimal Euler–Lagrange trajectory for the cytosolic calcium concentration $u_1(t)$. The horizontal reference line indicates the equilibrium value $u_1^*$. This figure shows how the rare opening of channels is accompanied by an excursion in cytosolic calcium

To assess the large-deviation prediction against the stochastic dynamics, we compare the Euler–Lagrange optimal path and its associated action with Monte Carlo simulations of the PDMP. At the trajectory level, we compare the optimal path with a conditioned rare ensemble extracted from the simulated paths, through its conditioned mean and a pointwise 10–90% band. This shows whether the variational minimizer captures the typical temporal profile followed by the stochastic system when the rare event occurs. At the probability level, we compare the action $S^*(T,\hat{x})$ with the empirical Monte Carlo rate $-\log (P_{\textrm{MC}})/N$, since the large-deviation principle predicts $P_{\textrm{MC}}$ has the exponential asymptotic form $\exp (-N S^*)$ up to subexponential prefactors. Thus, the comparison tests both the shape of the predicted rare trajectory and the exponential scaling of its probability.

**Fig. 3:**
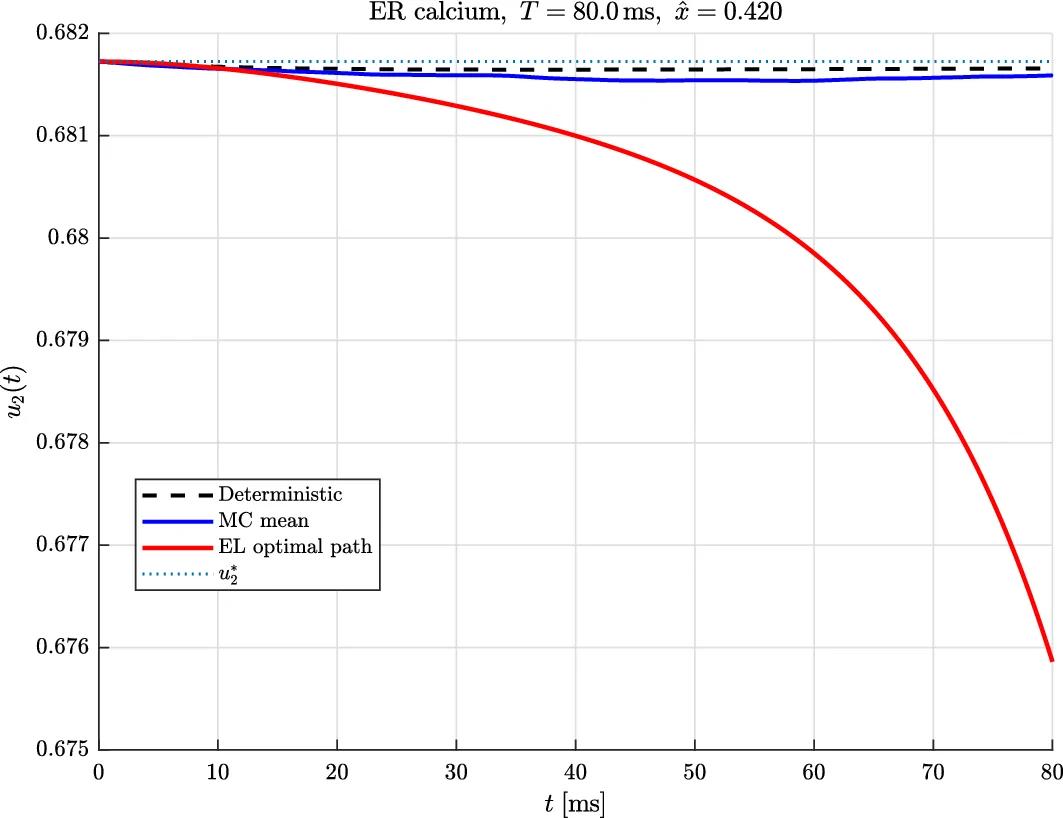
Comparison of the deterministic trajectory, the Monte Carlo mean trajectory, and the optimal Euler–Lagrange trajectory for the endoplasmic reticulum calcium concentration $u_2(t)$. The horizontal reference line indicates the equilibrium value $u_2^*$. This figure shows the complementary calcium dynamics in the endoplasmic reticulum during the rare-event excursion

**Fig. 4:**
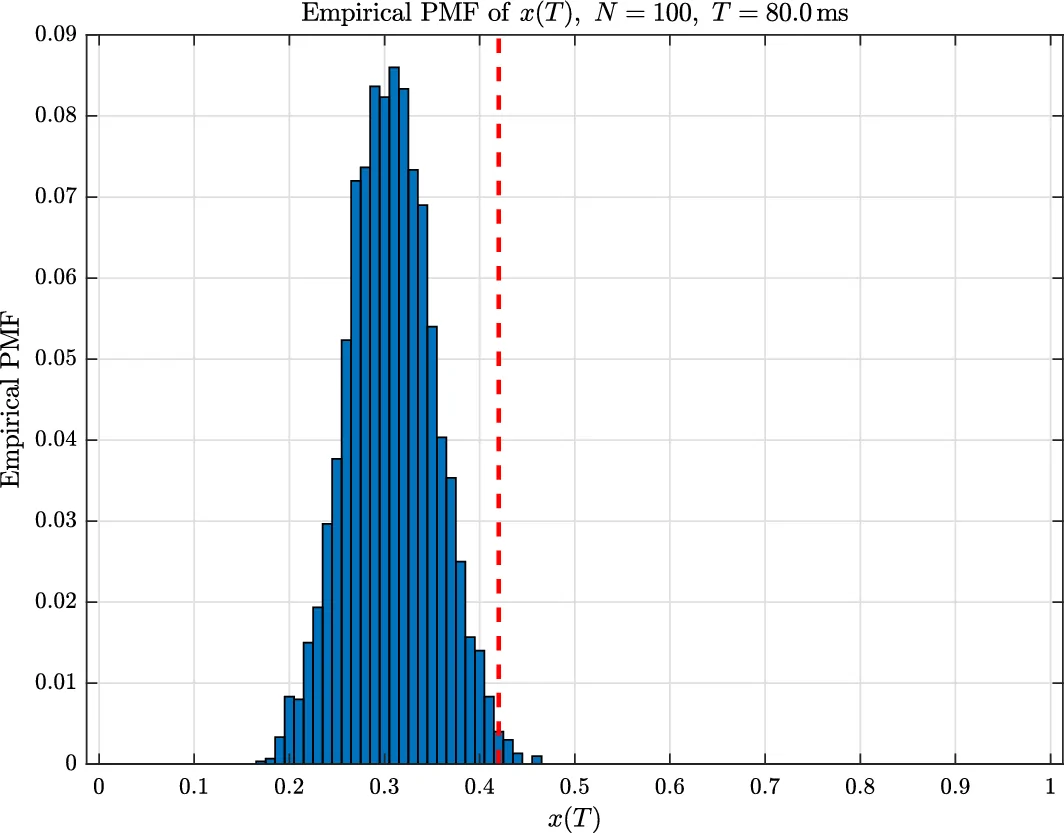
Empirical probability mass function of the final-time channel fraction *x*(*T*) on the lattice $\{0,1/N,\dots ,1\}$, obtained from the continuous-time PDMP Monte Carlo simulation. The vertical dashed line marks the target threshold $\hat{x}$. This figure displays directly the final-time distribution used to estimate the rare-event probability $\mathbb {P}(x(T)\ge \hat{x})$

**Fig. 5:**
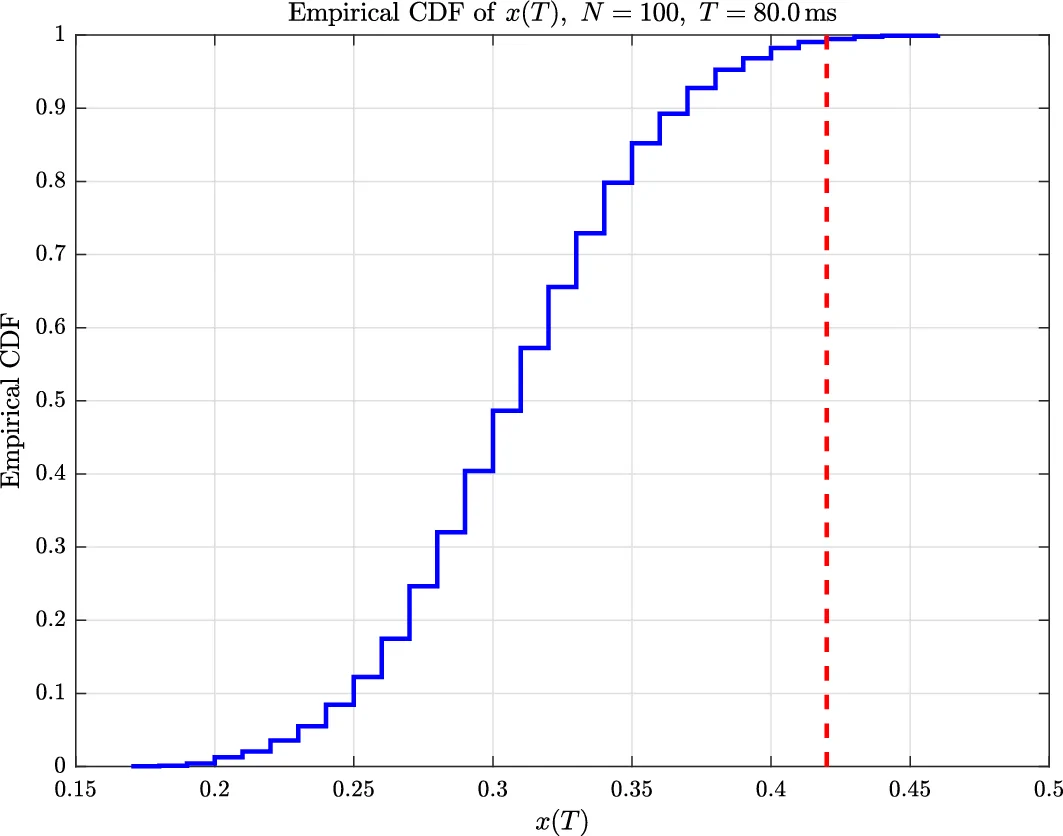
Empirical cumulative distribution function of the final-time channel fraction *x*(*T*), together with the threshold $\hat{x}$. This gives an alternative visualization of the final-time rare-event probability, since $\mathbb {P}(x(T)\ge \hat{x}) = 1 - F_{x(T)}(\hat{x}^{-})$

**Fig. 6:**
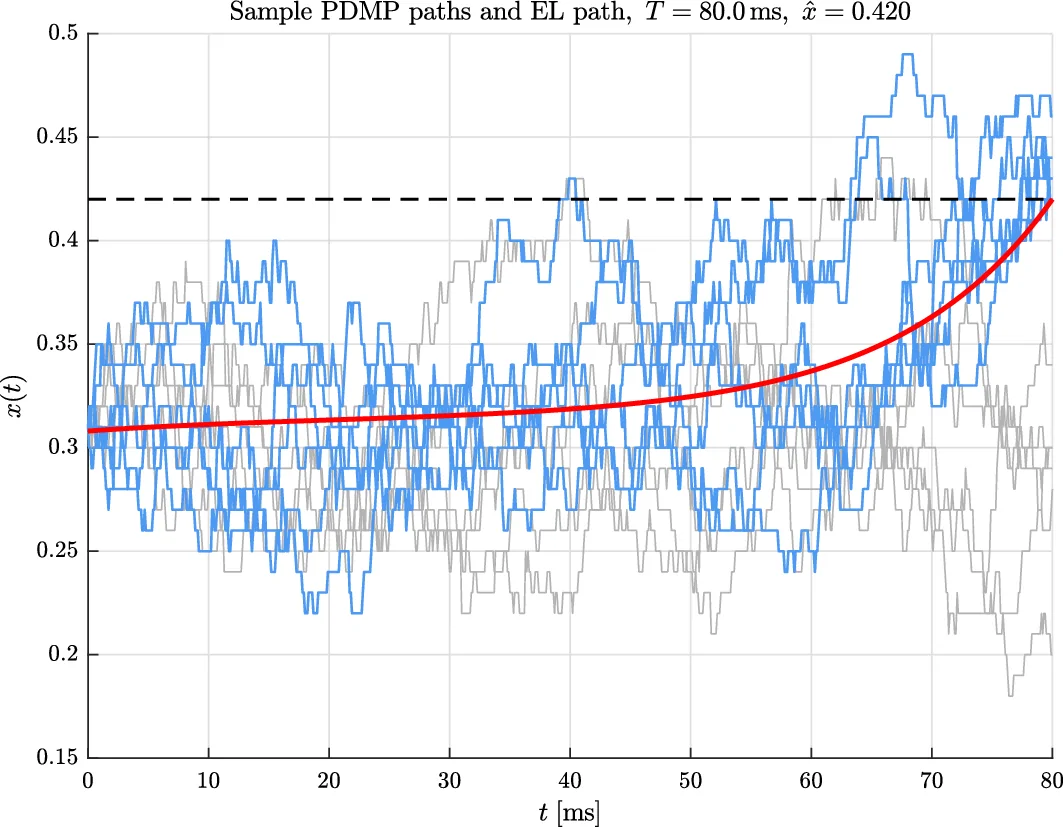
Representative stored PDMP sample paths for *x*(*t*), together with the optimal Euler–Lagrange path. Paths with $x(T)<\hat{x}$ are shown separately from those with $x(T)\ge \hat{x}$, and the threshold $\hat{x}$ is indicated by a horizontal dashed line. This figure illustrates how the optimal path sits relative to individual stochastic realizations

**Fig. 7:**
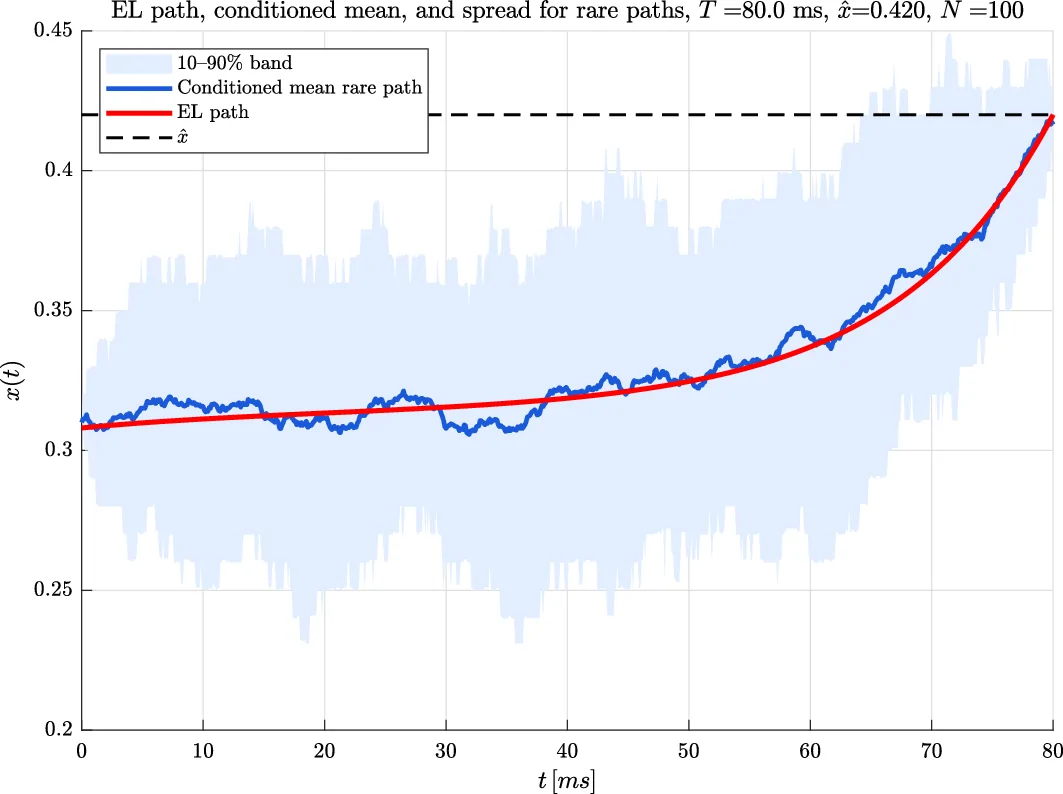
Comparison between the optimal Euler–Lagrange path and the conditioned rare ensemble for *x*(*t*). The optimal path is shown together with the conditioned mean rare path and the pointwise 10%–90% band of the conditioned stochastic trajectories. The conditioning is performed on PDMP trajectories whose final value *x*(*T*) lies near the lattice point closest to $\hat{x}$. This gives the most direct comparison between the large-deviation minimizer and the stochastic rare-event ensemble

**Fig. 8:**
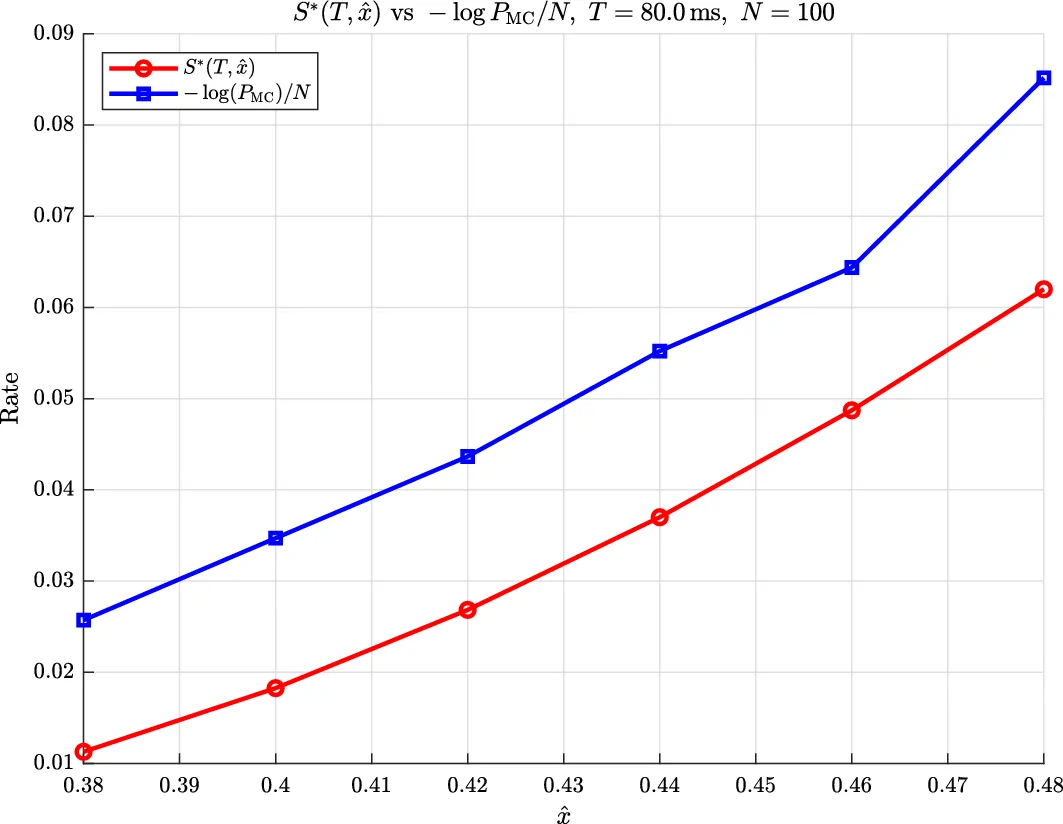
Comparison of the optimal action $S^*(T,\hat{x})$ and the Monte Carlo rate $-\log (P_{\textrm{MC}})/N$ as functions of the target level $\hat{x}$, for fixed *T* and *N*. Agreement between these two quantities is the expected large-deviation signature for the final-time rare event $\{x(T)\ge \hat{x}\}$

The numerical results are summarized through the following plots. The first group compares the deterministic solution, the Monte Carlo mean of the PDMP, and the optimal Euler–Lagrange trajectory for the variables *x*(*t*), $u_1(t)$, and $u_2(t)$, thereby showing the rare excursion away from equilibrium in both the channel fraction and the calcium concentrations (Figs. 1, 2 and 3). The next group examines the final-time statistics of the stochastic system through the empirical probability mass function and cumulative distribution function of *x*(*T*), with the target level $\hat{x}$ indicated explicitly (Figs. 4 and 5). We then display representative PDMP sample paths together with the optimal path, in order to visualize how the large-deviation minimizer sits relative to individual stochastic realizations (Fig. 6). We also compare the optimal trajectory with the conditioned rare ensemble, showing the conditioned mean path and a pointwise 10–90% band for trajectories whose endpoint lies near the lattice point closest to $\hat{x}$ (Fig. 7). Finally, we plot $S^*(T,\hat{x})$ against $-\log (P_{\textrm{MC}})/N$ as a function of $\hat{x}$, to assess the large-deviation prediction quantitatively (Fig. 8).

### Discussion and further work

We have demonstrated that large deviation theory can be used to compute the most likely trajectory followed by *N* stochastic channels / diffusing calcium in making the spark to wave transition (Dupont et al. 2016). We were able to verify through repeated stochastic simulations that the rare stochastic transitions corresponded closely to the predictions of large deviation theory. This model is relatively simple, particularly insofar as the calcium concentration is spatially homogeneous. In a subsequent work, we will employ a spatially-distributed model of the calcium to compute the optimal trajectories. Indeed there is excellent experimental evidence that calcium waves are spatially-heterogeneous fronts that propagate throughout the cell before subsiding (Dupont et al. 2016). For this reason, several scholars have developed spatially-extended stochastic models to explain the spatially-heterogeneous structure. Perhaps the most well-known is the fire–diffuse–fire framework has been introduced by Keizer, Keizer et al. (1998); Dawson et al. (2002). The fire–diffuse–fire model consists of an array of point-source release sites (corresponding to the channels) coupled by continuously diffusing particles such as $Ca^{2+}$ and IP3. The original papers concerned cardiac myocytes, and the point-sources are RyR receptors embedded in the Sarcoplasmic Reticulum. In the original papers (Keizer et al. 1998; Dawson et al. 2002), the point sources were modelled deterministically using Ordinary Differential Equations, and the diffusing particles satisfy a reaction-diffusion Partial Differential Equation. In later work, Keizer, Smith and co-workers modeled the point-sources as Poisson Processes and performed stochastic simulations - obtaining strong numerical evidence for noise-induced waves.

## Proofs

To prove the upper bound of Theorem 1.1, we apply the Contraction Principle (Dembo and Zeitouni 1998) to the large deviation principle for the independent system. In other words, we are going to perform a change of variable, which will transfer known results for the large deviations of independent poisson processes (Weiss and Schwartz 1995, Chapter 6). We first recount the large deviations for independent Poisson processes (recalling the definition of $\lbrace y_{\alpha }(t) \rbrace _{\alpha \in \mathscr {R}}$ in Eq. (20)).

### Theorem 4.1

The sequence of probability laws of $(y_{\alpha })_{\alpha \in \mathscr {R}} \in \tilde{\mathcal {D}}([0,\infty ),\mathbb {R}^+)^M$ satisfy a large deviation principle with good rate function.

That is, for $\mathcal {O},\mathcal {A} \subset \tilde{\mathcal {D}}([0,\infty ),\mathbb {R}^+)^M$, with $\mathcal {O}$ open and $\mathcal {A}$ closed,

102 $$\begin{aligned} \underset{N\rightarrow \infty }{\overline{\lim }}N^{-1}\log \mathbb {P}\big ( y \in \mathcal {A} \big )&\le - \inf _{\alpha \in \mathcal {A}}\mathcal {I}(\alpha ) \end{aligned}$$

103 $$\begin{aligned} \underset{N\rightarrow \infty }{\underline{\lim }}N^{-1}\log \mathbb {P}\big ( y \in \mathcal {O} \big )&\ge - \inf _{\alpha \in \mathcal {O}}\mathcal {I}(\alpha ) , \end{aligned}$$

and the rate function is

104 $$\begin{aligned} \mathcal {I}(y)&= \left\{ \begin{array}{c} \infty \text { in the case that }y_{\alpha } \text { is not absolutely continuous for some }\alpha \in \mathscr {R} \\ \sum _{\alpha \in \mathscr {R}} \int _0^\infty \ell \big ( \dot{y}_{\alpha }(r) \big )dr \text { otherwise, } \end{array}\right. \end{aligned}$$

105 $$\begin{aligned} \ell (r)&= r\log r -r +1. \end{aligned}$$

Furthermore the level sets of $\mathcal {I}$ are compact.

### Proof

The large deviations principle for arbitrarily long finite time intervals is proved in (Weiss and Schwartz, 1995, Chapter 5), with corresponding rate function

106 $$\begin{aligned} \mathcal {I}(y) = \sum _{\alpha \in \mathscr {R}} \int _0^{\infty } \ell \big ( \dot{y}_{\alpha }(r) \big )dr. \end{aligned}$$

□

The first result that we must prove is that the large deviations principle must hold for sets with reaction rates bounded away from zero.

### Lemma 4.2

Suppose that $\mathcal {O},\mathcal {A} \subseteq \Upsilon$, with $\mathcal {O}$ open and $\mathcal {A}$ closed, are such that for any T>0,

107 $$\begin{aligned} \inf _{ (z,x,u) \in \mathcal {A} \cup \mathcal {O}} \inf _{\alpha \in \mathscr {R}} \inf _{s\in [0,T]} \lambda _\alpha (x(s),u(s)) > 0. \end{aligned}$$

Then the sequence of probability laws of $\big ( z,x,u \big )$ satisfy a large deviation principle on the space Υ, i.e.

108 $$\begin{aligned} \underset{N\rightarrow \infty }{\overline{\lim }}N^{-1}\log \mathbb {P}\big ( (z,x,u) \in \mathcal {A} \big )&\le - \inf _{\beta \in \mathcal {A}}\mathcal {J}(\beta ) \end{aligned}$$

109 $$\begin{aligned} \underset{N\rightarrow \infty }{\underline{\lim }}N^{-1}\log \mathbb {P}\big ( (z,x,u) \in \mathcal {O} \big )&\ge - \inf _{\beta \in \mathcal {O}}\mathcal {J}(\beta ). \end{aligned}$$

$\mathcal {J}$ is lower-semi-continuous and the level sets of $\mathcal {J}$ are compact.

### Proof

We start with the upper bound. Define a mapping $\Psi : \Upsilon \rightarrow \tilde{\mathcal {D}}([0,\infty ),\mathbb {R}^+)^M$ as follows. For any $(\tilde{z},\tilde{x},\tilde{u}) \in \Upsilon$, define

110 $$\begin{aligned} \Lambda _{\alpha }(t) = \int _0^t \lambda _{\alpha }(\tilde{x}(s),\tilde{u}(s)) ds. \end{aligned}$$

Let $\Lambda ^{-1}_{\alpha } \in \tilde{\mathcal {D}}([0,\infty ),\mathbb {R}^+)$ be such that

111 $$\begin{aligned} \Lambda ^{-1}_{\alpha }(t) = \inf \big \lbrace s\ge 0 \; : \Lambda _{\alpha }(s) = t \big \rbrace . \end{aligned}$$

Note that, since $\inf _{s\in [0,t]}\lambda _{\alpha }(x(s),u(s)) > 0$, it must be that $\Lambda ^{-1}_{\alpha }$ is the function inverse of $\Lambda _{\alpha }$. Write

112 $$\begin{aligned} \tau _{\alpha } = \lim _{t\rightarrow \infty } \Lambda _{\alpha }(t) , \end{aligned}$$

and note that $\tau _{\alpha }$ could be ∞. We define $\Psi (\tilde{z},\tilde{x},\tilde{u}) = \big ( w_{\alpha } \big )_{\alpha \in \mathscr {R}}$, where for any $t < \tau _{\alpha }$,

113 $$\begin{aligned} w_{\alpha }(t) = \tilde{z}_{\alpha }\big ( \Lambda ^{-1}_{\alpha }(t) \big ). \end{aligned}$$

Notice that Ψ(z,x,u)=y (recall that $y_{\alpha }(t) = N^{-1} Y_{\alpha }(Nt)$ and (*z*, *x*, *u*) are the stochastic processes in Sect. 2). For L>0, let $\mathcal {K}_L$ be the compact set from Lemma 4.6, such that

114 $$\begin{aligned} \underset{N\rightarrow \infty }{\overline{\lim }} N^{-1}\log \mathbb {P}\big ( (z,x,u) \notin \mathcal {K}_L \big ) \le - L. \end{aligned}$$

Since Ψ is continuous, and $\mathcal {A} \cap \mathcal {K}_L$ is compact, it must be that $\Psi \big (\mathcal {A}\cap \mathcal {K}_L\big )$ is also compact. Hence

115 $$\begin{aligned} \underset{N\rightarrow \infty }{\overline{\lim }}N^{-1}\log \mathbb {P}\big ( (z,x,u) \in \mathcal {A} \cap \mathcal {K}_L \big )&= \underset{N\rightarrow \infty }{\overline{\lim }}N^{-1}\log \mathbb {P}\big ( y \in \Psi (\mathcal {A} \cap \mathcal {K}_L) \big ) \nonumber \\&\le - \inf _{\beta \in \Psi (\mathcal {A} \cap \mathcal {K}_L)}\mathcal {I}(\beta ), \end{aligned}$$

using the LDP for the counting processes in Theorem 4.1. We hence find that

116 $$\begin{aligned} \underset{N\rightarrow \infty }{\overline{\lim }}N^{-1}\log \mathbb {P}\big ( (z,x,u) \in \mathcal {A} \big )\nonumber \\ \le \max \bigg \lbrace \underset{N\rightarrow \infty }{\overline{\lim }}N^{-1}\log \mathbb {P}\big ( (z,x,u) \in \mathcal {A} \cap \mathcal {K}_L \big ), \underset{N\rightarrow \infty }{\overline{\lim }}N^{-1}\log \mathbb {P}\big ( (z,x,u) \notin \mathcal {K}_L \big ) \bigg \rbrace \nonumber \\ \le \max \bigg \lbrace - L, - \inf _{\beta \in \Psi (\mathcal {A} \cap \mathcal {K}_L)}\mathcal {I}(\beta )\bigg \rbrace . \end{aligned}$$

Furthermore, the definition of the rate function $\mathcal {J}$ implies that

117 $$\begin{aligned} \inf _{\beta \in \Psi (\mathcal {A}\cap \mathcal {K}_L)}\mathcal {I}(\beta ) = \inf _{\beta \in \mathcal {A}\cap \mathcal {K}_L}\mathcal {J}(\beta ). \end{aligned}$$

Taking $L\rightarrow \infty$, and using the lower-semicontinuity of $\mathcal {I}$, we obtain (108). The lower bound is proved further below in Lemma 4.3.

□

### Lemma 4.3

Suppose that $\mathcal {O} \subset \Upsilon$ is open. Then

118 $$\begin{aligned} \underset{N\rightarrow \infty }{\underline{\lim }}N^{-1}\log \mathbb {P}\big ( (z,x,u) \in \mathcal {O} \big ) \ge - \inf _{\alpha \in \mathcal {O}}\mathcal {J}(\alpha ). \end{aligned}$$

### Proof

If $\inf _{(\tilde{z},\tilde{x},\tilde{u}) \in \mathcal {O}}\mathcal {J}(\tilde{z},\tilde{x},\tilde{u}) = \infty$, then the Lemma is immediate. Otherwise, we suppose that

119 $$\begin{aligned} \inf _{(\tilde{z},\tilde{x},\tilde{u}) \in \mathcal {O}}\mathcal {J}(\tilde{z},\tilde{x},\tilde{u} ) < \infty , \end{aligned}$$

and let $(\tilde{z},\tilde{x},\tilde{u})$ be an arbitrary member of $\mathcal {O}$ that is such that

120 $$\begin{aligned} \mathcal {J}(\tilde{z},\tilde{x},\tilde{u}) < \infty . \end{aligned}$$

Since $(\tilde{z},\tilde{x},\tilde{u})$ is an arbitrary member of $\mathcal {O}$ satisfying (120), it suffices that we prove that

121 $$\begin{aligned} \underset{N\rightarrow \infty }{\underline{\lim }}N^{-1}\log \mathbb {P}\big ( (z,x,u) \in \mathcal {O} \big ) \ge - \mathcal {J}(\tilde{z},\tilde{x},\tilde{u}) . \end{aligned}$$

For a>0, let $\mathcal {V}_a \subseteq \tilde{\mathcal {D}}\big ( [0,\infty ), \mathbb {R} \big )^{\mathscr {R}}$ consist of all $(y_{\alpha })_{\alpha \in \mathscr {R}}$ such that (i) $t \mapsto y_{\alpha }(t)$ is continuous and piecewise differentiable,with a finite number of points where it is not differentiable over any bounded time interval, and the derivative is Lipschitz with Lipschitz constant less than or equal to *a*, (ii) $y_{\alpha }(0) = 0$, (iii) $y_{\alpha }$ is non-decreasing.

Since $\mathcal {J}(\tilde{z},\tilde{x},\tilde{u}) < \infty$, it must be that $t \mapsto \tilde{z}_{\alpha }(t)$ is absolutely continuous. It can thus be uniformly approximated by functions in $\mathcal {V}_a$. We thus find that for any ϵ>0 there exists $a \in \mathbb {Z}^+$ and $z^{(\epsilon )} \in \mathcal {V}_a$ such that

122 $$\begin{aligned} \sum _{\alpha \in \mathscr {R}} d_{\infty }^{\circ }\big ( z^{(\epsilon )}_{\alpha }, \tilde{z}_{\alpha } \big ) \le \epsilon . \end{aligned}$$

We can further assume that $z^{(\epsilon )}$ is piecewise-linear, i.e. it is such that for $\delta \ll 1$, all positive integers *b* and all $\eta \in [0,1]$,

123 $$\begin{aligned} z^{(\epsilon )}_{\alpha }\big ( \eta b \delta + (1-\eta ) (b+1)\delta \big ) = \eta z_{\alpha }(b\delta ) + (1-\eta )z_{\alpha }\big ( (b+1)\delta ) \big ) \end{aligned}$$

Now define

124 $$\begin{aligned} x^{(\epsilon )}(t) =&\hat{x}_0 + \sum _{\alpha \in \mathscr {R}} z_{\alpha }^{(\epsilon )}(t) \xi _{\alpha } \end{aligned}$$

125 $$\begin{aligned} \frac{du^{(\epsilon )}}{dt} =&A\big ( u^{(\epsilon )}(t) , x^{(\epsilon )}(t) \big ) \end{aligned}$$

and $u^{(\epsilon )}(0) = \hat{u}_0$. Clearly $\big ( z^{(\epsilon )}, x^{(\epsilon )}, u^{(\epsilon )} \big ) \in \Upsilon$, and thanks to (122) and Lemma 1.2 it holds that for any t>0,

126 $$\begin{aligned} \sup _{j \le d} d^{\circ }_t ( x^{(\epsilon )}_j , \tilde{x}_j ) + \sup _{s\le t} \Vert u^{(\epsilon )}(s) - \tilde{u}(s) \Vert \le 2c_t \epsilon . \end{aligned}$$

Write $y^{(\epsilon )} = \Psi (z^{(\epsilon )}, x^{(\epsilon )}, u^{(\epsilon )})$. It thus follows from Lemma 4.5 (below) that, as long as ϵ is sufficiently small, there exists ι>0 such that

$$ \bigg \lbrace \sup _{\alpha \in \mathscr {R}} d^{\circ }_{\infty }\big (y_{\alpha }, y^{(\epsilon )}_{\alpha }\big ) < \iota \bigg \rbrace \subseteq \bigg \lbrace (z,x,u) \in \mathcal {O} \bigg \rbrace . $$

We therefore find that

127 $$\begin{aligned} \mathbb {P}\big ( (z,x,u) \in \mathcal {O} \big ) \ge&\mathbb {P}\big ( \sup _{\alpha \in \mathscr {R}} d^{\circ }_{\infty }\big (y_{\alpha } , y^{(\epsilon )}_{\alpha }\big ) < \iota \big ) . \end{aligned}$$

The large deviations lower bound in Theorem 4.1 implies that

128 $$\begin{aligned} \underset{N\rightarrow \infty }{\underline{\lim }} N^{-1}\log \mathbb {P}\big (&\sup _{\alpha \in \mathscr {R}} d^{\circ }_{\infty }\big (y_{\alpha } , y^{(\epsilon )}_{\alpha }\big )< \delta \big ) \ge - \inf \big \lbrace \mathcal {I}(y) \; : \; \sup _{\alpha \in \mathscr {R}} d^{\circ }_{\infty }\big (y_{\alpha } , y^{(\epsilon )}_{\alpha }\big ) < \delta \big \rbrace \end{aligned}$$

129 $$\begin{aligned} \ge&- \mathcal {I}\big (y^{(\epsilon )} \big ) \nonumber \\ =&- \sum _{\alpha \in \mathscr {R}}\int _{\mathbb {R}^+ } \ell \bigg ( \dot{z}^{(\epsilon )}_{\alpha }(r) / \lambda _{\alpha }(x^{(\epsilon )}(r),u^{(\epsilon )}(r)) \bigg )\lambda _{\alpha }(x^{(\epsilon )}(r),u^{(\epsilon )}(r)) dr \end{aligned}$$

using the expression for the rate function in Eq. (29). Since $\tilde{z}_{\alpha }$ must be uniformly continuous over finite time intervals, it must hold that for any T>0,

130 $$\begin{aligned} \lim _{\epsilon \rightarrow 0^+} \sup _{\alpha \in \mathscr {R}} \sup _{t\le T} \big | z^{(\epsilon )}_{\alpha }(t) - \tilde{z}_{\alpha }(t) \big | = 0, \end{aligned}$$

and therefore

131 $$\begin{aligned} \lim _{\epsilon \rightarrow 0^+} \sup _{ j \le d} \sup _{t\le T} \big | x^{(\epsilon )}_{j}(t) - \tilde{x}_{j}(t) \big |&= 0 \end{aligned}$$

132 $$\begin{aligned} \lim _{\epsilon \rightarrow 0^+} \sup _{t\le T} \big \Vert u^{(\epsilon )}(t) - \tilde{u}(t) \big \Vert&= 0 . \end{aligned}$$

We finally claim that for any T>0

133 $$\begin{aligned} \lim _{\iota \rightarrow 0^+}\lim _{\epsilon \rightarrow 0^+} \int _0^T \chi \bigg \lbrace \sup _{\alpha \in \mathscr {R}} \big | \dot{\tilde{z}}_{\alpha }(t) - \dot{z}_{\alpha }^{(\epsilon )}(t) \big | \ge \iota \bigg \rbrace dt = 0. \end{aligned}$$

Indeed if (133) did not hold, then it would be impossible that (130) holds.

We thus find that for any T,L>0,

134 $$\begin{aligned} &  \lim _{\epsilon \rightarrow 0^+} \sum _{\alpha \in \mathscr {R}}\int _{0}^T \ell \bigg ( \dot{z}^{(\epsilon )}_{\alpha }(r) / \lambda _{\alpha }(x^{(\epsilon )}(r),u^{(\epsilon )}(r)) \bigg )\lambda _{\alpha }(x^{(\epsilon )}(r),u^{(\epsilon )}(r)) dr\nonumber \\ &  \ge \sum _{\alpha \in \mathscr {R}}\int _{0 }^T \chi \bigg \lbrace \sup _{\alpha \in \mathscr {R}} \dot{\tilde{z}}_{\alpha }(r) \le L \;, \; \left\| \tilde{u}(r) \right\| \le L \;, \; \left\| \tilde{x}(r) \right\| \nonumber \\ &  \quad \le L \bigg \rbrace \ell \bigg ( \dot{\tilde{z}}_{\alpha }(r) / \lambda _{\alpha }( \tilde{x}(r),\tilde{u}(r)) \bigg )\lambda _{\alpha }(\tilde{x}(r),\tilde{u}(r)) dr. \end{aligned}$$

Taking $T,L \rightarrow \infty$, since the integrand is non-negative, the RHS converges to $\mathcal {J}(\tilde{z},\tilde{x},\tilde{u})$. We have therefore proved (121). □

### Lemma 4.4

Suppose that $y \in \mathcal {V}_a$ for some a>0. Then there exists a unique $(z_{\alpha }(t))_{\alpha \in \mathscr {R}}$ such that, writing the derivative of $y_{\alpha }$ as $\dot{y}_{\alpha }$ (by definition of $\mathcal {V}_a$, the derivative does not exist at only a finite number of points),

135 $$\begin{aligned} \frac{dz_{\alpha }}{dt} =&\dot{y}_{\alpha }\big ( \Lambda _{\alpha }( t) \big ) \lambda _{\alpha }(x(t) , u(t))\end{aligned}$$

136 $$\begin{aligned} \frac{d}{dt} \Lambda _{\alpha }( t ) =&\lambda _{\alpha }(x(t) , u(t)) \end{aligned}$$

137 $$\begin{aligned} \frac{du}{dt} =&A\big ( u(t), x(t) \big ). \end{aligned}$$

### Proof

We have assumed at the start of the paper that every function is globally Lipschitz. The existence and uniqueness thus follows from the Picard-Lindelof Theorem over each interval over which $\dot{y}$ exists. Because for each time interval [0, *T*], there are only a finite number of points where $\dot{y}$ does not exist, the existence and uniqueness must hold for arbitrarily long times. □

### Lemma 4.5

Let $(\tilde{z},\tilde{x},\tilde{u}) \in \mathcal {V}_a$ for some a>0, and assume that

138 $$\begin{aligned} \mathcal {J}(\tilde{z},\tilde{x},\tilde{u}) < \infty \end{aligned}$$

Write $\tilde{y} = \Psi (\tilde{z},\tilde{x},\tilde{u})$. For any δ>0, there exists ϵ>0 such that if

139 $$\begin{aligned} \sum _{\alpha \in \mathscr {R}} d^\circ _{\infty }( y_{\alpha }, \tilde{y}_{\alpha }) < \epsilon \end{aligned}$$

then necessarily (as long as *n* is large enough),

140 $$\begin{aligned} \sum _{\alpha \in \mathscr {R}} d^\circ _{\infty }( z_{\alpha }, \tilde{z}_{\alpha }) < \delta . \end{aligned}$$

### Proof

Define $\big ( \hat{z}_{\alpha } \big )_{\alpha \in \mathscr {R}}$ to be such that

141 $$\begin{aligned} \hat{z}_{\alpha }(t) = \tilde{y}_{\alpha }\bigg ( \int _0^t \lambda _{\alpha }\big ( x(s), u(s) \big ) ds \bigg ), \end{aligned}$$

and note that necessarily

142 $$\begin{aligned} \tilde{z}_{\alpha }(t) = \tilde{y}_{\alpha }\bigg ( \int _0^t \lambda _{\alpha }\big ( \tilde{x}(s), \tilde{u}(s) \big ) ds \bigg ). \end{aligned}$$

Now $t \mapsto \tilde{y}_{\alpha }(t)$ is Lipschitz, with constant *a*, over the time interval [0, *a*] (by definition of $\mathcal {V}_a$). We thus find that for all t≤a,

143 $$\begin{aligned} \sup _{\alpha \in \mathscr {R}} \big | \tilde{z}_{\alpha }(t) - \hat{z}_{\alpha }(t) \big | \le a \int _0^t \sup _{\alpha \in \mathscr {R}} \big | \lambda _{\alpha }\big ( x(s), u(s) \big ) - \lambda _{\alpha }\big ( \tilde{x}(s), \tilde{u}(s) \big ) \big | ds . \end{aligned}$$

Now one readily checks that there is a constant C>0 such that

144 $$\begin{aligned}&\sup _{\alpha \in \mathscr {R}} \big | \lambda _{\alpha }\big ( x(s), u(s) \big ) - \lambda _{\alpha }\big ( \tilde{x}(s), \tilde{u}(s) \big ) \big | \le C\sup _{\alpha \in \mathscr {R}} \sup _{r\le s} \big | \tilde{z}_{\alpha }(r) - \hat{z}_{\alpha }(r) \big | \nonumber \\&+C \Vert \tilde{x}(0) - x(0) \Vert . \end{aligned}$$

Since $\mathcal {J}(\tilde{z}, \tilde{x}, \tilde{u}) < \infty$, it must be that $\tilde{x}(0) = \hat{x}_0$. Thanks to Gronwall’s Inequality, we thus find that there is a constant $\tilde{c}_a$ such that

145 $$\begin{aligned} \sup _{\alpha \in \mathscr {R}} \big | \tilde{z}_{\alpha }(t) - \hat{z}_{\alpha }(t) \big | \le \tilde{c}_a \left\| x(0) - \hat{x}_0 \right\| . \end{aligned}$$

By assumption, $\left\| x(0) - \hat{x}_0 \right\|$ goes to 0 uniformly as $n\rightarrow \infty$, and thus

146 $$\begin{aligned} \sup _{\alpha \in \mathscr {R}} \big | \tilde{z}_{\alpha }(t) - \hat{z}_{\alpha }(t) \big | \le \delta / 2, \end{aligned}$$

for all large enough *n*.

It remains to show that, as long as ϵ is small enough,,

147 $$\begin{aligned} \sum _{\alpha \in \mathscr {R}} d^{\circ }_{\infty }\big ( \hat{z}_{\alpha }, z_{\alpha } \big ) \le \delta / 2. \end{aligned}$$

Observe that there exists a differentiable function $\Upsilon _{\alpha }(t)$ such that $\hat{z}_{\alpha }(t):= \tilde{y}_{\alpha }\big ( \Upsilon _\alpha (t) \big )$ and $z_{\alpha }(t) = y_{\alpha }\big ( \Upsilon _{\alpha }(t) \big )$. Also let $\Lambda _{\alpha }(t)$ be a function such that

148 $$\begin{aligned} \tilde{d}^{\circ }_T\big ( y_{\alpha } , \tilde{y}_{\alpha } \big ) \le \sup \big | \tilde{y}_{\alpha }(t) - y_{\alpha }\big ( \Lambda _{\alpha }(t) \big ) \big | + \frac{\delta }{4}. \end{aligned}$$

Fix T>0. One can check that, over any interval [r,s]⊆[0,T] over which $t \mapsto \big ( \tilde{z}_{\alpha }(t) \big )_{\alpha \in \mathscr {R}}$ is differentiable, there exists a constant $C_{a,T} > 0$ such that

149 $$\begin{aligned}&\sup _{\alpha \in \mathscr {R}} \bigg | \frac{dz_{\alpha }}{dt} - \frac{d\hat{z}_{\alpha }}{dt} \bigg | \le C_{a,T} \big \lbrace \Vert u(t) - \tilde{u}(t) \Vert + \Vert x(t) - \tilde{x}(t) \Vert \nonumber \\&+ \sum _{\alpha \in \mathscr {R}}\big ( d^{\circ }_{t}\big ( \hat{z}_{\alpha }, z_{\alpha } \big ) + d^{\circ }_{\infty }\big ( \hat{z}_{\alpha }, \tilde{z}_{\alpha } \big ) \big ) \big \rbrace \end{aligned}$$

An application of Gronwall’s Inequality therefore implies that there must exist a constant $\tilde{C}_{a,T}$ such that

150 $$\begin{aligned} \sup _{\alpha \in \mathscr {R}} \bigg | \frac{dz_{\alpha }}{dt} - \frac{d\hat{z}_{\alpha }}{dt} \bigg | \le \tilde{C}_{a,T} d^{\circ }_{T}\big ( \hat{z}_{\alpha }, \tilde{z}_{\alpha } \big ). \end{aligned}$$

Now (as noted above) we can take $d^{\circ }_{T}\big ( \hat{z}_{\alpha }, \tilde{z}_{\alpha } \big )$ to be arbitrarily small by taking $\epsilon \rightarrow 0^+$. This implies the Lemma. □

The following exponential tightness result is a standard requirement for large deviation principles.

### Lemma 4.6

For any L>0, there exists a compact set $\mathcal {K}_{L} \subset \Upsilon$ such that

151 $$\begin{aligned} \underset{N\rightarrow \infty }{\overline{\lim }} N^{-1}\log \mathbb {P}\big ( (z,x,u) \notin \mathcal {K}_L \big ) \le - L. \end{aligned}$$

### Proof

For a positive integer a≥10, define

$$ t^{(a)}_b = \frac{b}{a}. $$

and then define the set $\mathcal {L}_{a} \subset \Upsilon$ to consist of all $(\tilde{z}, \tilde{x}, \tilde{u})$ such that

152 $$\begin{aligned} \sup _{b \le a^2 } \sup _{\alpha \in \mathscr {R}} \bigg \lbrace \tilde{z}_{\alpha }\big (t^{(a)}_{b+1}\big ) - \tilde{z}_{\alpha }\big (t^{(a)}_{b}\big ) \bigg \rbrace \le (\log \log a)^{-1}. \end{aligned}$$

Now using a union of events bound,

153 $$\begin{aligned} \mathbb {P}\big ( (z,x,u) \notin \mathcal {L}_a \big ) \le&\sum _{b=0}^{a^2-1} \sum _{\alpha \in \mathscr {R}} \mathbb {P}\bigg ( \tilde{z}_{\alpha }\big (t^{(a)}_{b+1}\big ) - \tilde{z}_{\alpha }\big (t^{(a)}_{b}\big ) > (\log \log a)^{-1}\bigg ) \end{aligned}$$

154 $$\begin{aligned} \le&a^2| \mathscr {R} | \mathbb {P}\bigg ( Y\big (CN / a \big ) > (\log \log a)^{-1} \bigg ) \end{aligned}$$

where *Y*(*t*) is a unit-intensity counting process, and using the fact that the intensity of $\tilde{z}_{\alpha }$ is upperbounded by *CN*. Thanks to Chebyshev’s Inequality, for a constant $b \in \mathbb {R}$,

155 $$\begin{aligned} \mathbb {P}\bigg ( Y\big (CN / a \big ) > N(\log \log a)^{-1} \bigg )&\le \mathbb {E}\bigg [ \exp \bigg ( b Y\big (CN / a \big ) - bN( \log \log a )^{-1} \bigg )\bigg ]\nonumber \\&= \exp \bigg ( \frac{CN}{a}\big \lbrace \exp (b)-1 \big \rbrace - bN ( \log \log a)^{-1} \bigg ). \end{aligned}$$

We differentiate the RHS with respect to *b* to find the optimal bound. Upon doing so, we find that $b = \log \big ( \frac{a \log a}{C} \big )$, and obtain that

156 $$\begin{aligned} \mathbb {P}\bigg ( Y\big (CN / a \big ) > N (\log \log a)^{-1} \bigg ) \le \exp \bigg ( -\frac{NC}{a} \ell \bigg (\frac{a}{C \log \log a} \bigg ) \bigg ). \end{aligned}$$

Notice that

157 $$\begin{aligned} \lim _{a\rightarrow \infty } \frac{C}{a} \ell \bigg (\frac{a}{C \log \log a} \bigg ) = \infty . \end{aligned}$$

We can therefore choose an increasing sequence $\lbrace a_i \rbrace _{i\ge 1}$ to be such that for all N≥1,

158 $$\begin{aligned} \mathbb {P}\bigg ( Y\big (CN / a_i \big ) > N(\log \log a_i)^{-1} \bigg ) \le \exp \big (-N i \big ). \end{aligned}$$

Let $\mathcal {K}_l \subset \Upsilon$ be the set

159 $$\begin{aligned} \mathcal {K}_l = \bigcap _{j\ge l} \mathcal {L}_{a_j}. \end{aligned}$$

A union-of-events bound then implies that

160 $$\begin{aligned} \mathbb {P}\big ( (z,x,u) \notin \mathcal {K}_i \big ) \le |\mathscr {R} |\sum _{j=i}^{\infty } j^2 \exp \big ( - Nj \big ). \end{aligned}$$

Hence for any l>0, for large enough *i* we find that for all N≥1,

161 $$\begin{aligned} N^{-1} \log \mathbb {P}\big ( (z,x,u) \notin \mathcal {K}_i \big ) \le - l. \end{aligned}$$

It remains to prove that $\mathcal {K}_i$ is compact. It is immediate from the construction of $\mathcal {K}_i$ that for $(z,x,u) \in \mathcal {K}_i$, the temporal fluctuations of *z* and *x* can be uniformly controlled over any finite time interval. Since $z_{\alpha }(t)$ is non-decreasing, this implies compactness in the Skorohod Topology (Billingsley 1999). *x* must also be compact, thanks to the identity

$$ \sum _{\alpha \in \mathscr {R}} z_{\alpha }(t) \zeta _{\alpha } = x(t) -x(0). $$

Finally, it is easy to check that *u*(*t*) must be uniformly continuous, and therefore compact. □

We finally note the proof of Lemma 1.2.

### Proof

Suppose that $$(z,x,u), (\tilde{z},\tilde{x}, \tilde{u}) \in \Upsilon $$.

It is clear from the definition of *x* that there is a universal constant *C* such that for all s≥0,

162 $$\begin{aligned} \left\| x(s) - \tilde{x}(s) \right\| \le C\sup _{\alpha \in \mathscr {R}}| z_{\alpha }(s) - \tilde{z}_{\alpha }(s) | +\left\| x(0) - \tilde{x}(0) \right\| . \end{aligned}$$

Also, thanks to our assumption on the growth of *u*(*t*) in Eq. (13),

163 $$\begin{aligned} \frac{d}{dt} \Vert u(t) \Vert&\le C\big ( 1 + \Vert u(t) \Vert \big )\end{aligned}$$

164 $$\begin{aligned} \frac{d}{dt} \Vert \tilde{u}(t) \Vert&\le C\big ( 1 + \Vert \tilde{u}(t) \Vert \big ). \end{aligned}$$

Gronwall’s Inequality now implies that

165 $$\begin{aligned} \Vert u(t) \Vert \le \big ( Ct + \Vert u(0) \Vert \big ) \exp \big ( tC \big )\end{aligned}$$

166 $$\begin{aligned} \Vert \tilde{u}(t) \Vert \le \Vert u(0) \Vert \exp \big ( tC \big ). \end{aligned}$$

This in turn implies that there is a constant $\tilde{C}$ such that

167 $$\begin{aligned} \sup _{t\le T}\big \Vert A\big ( u(t), x(t) \big ) \big \Vert&\le \tilde{C} \end{aligned}$$

168 $$\begin{aligned} \sup _{t\le T}\big \Vert A\big ( \tilde{u}(t), \tilde{x}(t) \big ) \big \Vert&\le \tilde{C} . \end{aligned}$$

We now find that there is a universal constant $c_t$ such that

169 $$\begin{aligned} \left\| {} \int _0^t A(u(s), x(s)) ds - \int _0^t A(\tilde{u}(s), \tilde{x}(s)) ds \right\| {} \le c_t \int _0^t \Vert {} u(s) - \tilde{u}(s) \Vert {} ds + c_t d_\infty ^\circ (x, \tilde{x}).\end{aligned}$$

Since $u(0) = \tilde{u}(0)$, it follows from Gronwall’s Inequality that

$$\sup _{s \le t} \Vert  u(s) - \tilde{u}(s) \Vert  \le c_t \exp (tc_t) d_\infty ^\circ (x, \tilde{x}). $$

In light of (162), we can conclude that $d_\infty ^\circ (x, \tilde{x}) \rightarrow 0$ whenever $\Vert {} x(0) - \tilde{x}(0) \Vert {} \rightarrow 0$ and

$$\sum _{\alpha \in \mathscr {R}} d_\infty ^\circ (z_\alpha , \tilde{z}_\alpha ) \rightarrow 0.$$

We thus find that for any t>0,

$$\sup _{s \le t} \Vert  u(s) - \tilde{u}(s) \Vert  \rightarrow 0,$$

as well.

## Data Availability

There is no associated Data with this paper.
